# Punctuated Distribution of Recombination Hotspots and Demarcation of Pericentromeric Regions in *Phaseolus vulgaris* L.

**DOI:** 10.1371/journal.pone.0116822

**Published:** 2015-01-28

**Authors:** Mehul S. Bhakta, Valerie A. Jones, C. Eduardo Vallejos

**Affiliations:** 1 Horticultural Sciences Department, University of Florida, Gainesville, Florida, United States of America; 2 Plant Molecular and Cellular Biology Program, University of Florida, Gainesville, Florida, United States of America; University of Idaho, UNITED STATES

## Abstract

High density genetic maps are a reliable tool for genetic dissection of complex plant traits. Mapping resolution is often hampered by the variable crossover and non-crossover events occurring across the genome, with pericentromeric regions (pCENR) showing highly suppressed recombination rates. The efficiency of linkage mapping can further be improved by characterizing and understanding the distribution of recombinational activity along individual chromosomes. In order to evaluate the genome wide recombination rate in common beans (*Phaseolus vulgaris* L.) we developed a SNP-based linkage map using the genotype-by-sequencing approach with a 188 recombinant inbred line family generated from an inter gene pool cross (Andean x Mesoamerican). We identified 1,112 SNPs that were subsequently used to construct a robust linkage map with 11 groups, comprising 513 recombinationally unique marker loci spanning 943 cM (LOD 3.0). Comparative analysis showed that the linkage map spanned >95% of the physical map, indicating that the map is almost saturated. Evaluation of genome-wide recombination rate indicated that at least 45% of the genome is highly recombinationally suppressed, and allowed us to estimate locations of pCENRs. We observed an average recombination rate of 0.25 cM/Mb in pCENRs as compared to the rest of genome that showed 3.72 cM/Mb. However, several hot spots of recombination were also detected with recombination rates reaching as high as 34 cM/Mb. Hotspots were mostly found towards the end of chromosomes, which also happened to be gene-rich regions. Analyzing relationships between linkage and physical map indicated a punctuated distribution of recombinational hot spots across the genome.

## Introduction

Molecular marker-based linkage maps have been used to connect the phenotype to the genotype using classical forward genetics. More specifically, these maps have been used to position genes in the genome, genetically deconstruct complex traits, and initiate map-based cloning projects. Linkage map construction and DNA marker technology have evolved dramatically from the use of isozymes, to hybridization-based detection of DNA, and to a great variety of PCR-based methodologies [1], culminating now in the incorporation of Next-Generation Sequencing (NGS) technologies like restriction-associated DNA sequencing (RADseq) [2] and genotyping-by-sequencing [3].

Mapping resolution of genes controlling simple or complex phenotypes is expected to increase with map density. However, a dense linkage map doesn’t always guarantee high resolution mapping at the molecular level because the probability of a crossover event is not uniform along chromosomes [4]. At one extreme are the pericentromeric regions (pCENR) where recombination is highly suppressed [5], and at the other extreme are the hotspots of recombination, which have been detected in plants using a population genetics approach [6], and a targeted approach [7]. Recombination is part of the apparent paradox of the need for chromosome breakage, through the introduction of double strand breaks (DSB) [8], to ensure the integrity of their transmission during meiosis. DSBs are repaired through homologous recombination resulting in either a crossover (CO), or a non-crossover (NCO) event, although the exact mechanisms controlling these outcomes have not yet been fully characterized [9]. There is clear evidence that the ratio of NCO over to CO is under genetic control [10, 11, 12]. Furthermore, the sites of CO/NCO events appear to occur at high frequencies at the 5’ end of CDS and at exons rather than introns [6, 7]. Furthermore, evidence from *Arabidopsis* indicates a strong association between recombination hotspots and certain chromatin states around the start of transcription of some genes [13]. Although linkage analysis doesn’t provide the resolution for recombination hotspots afforded by pollen-typing [7] or deep sequencing of diverse populations [6], it can still provide useful information for genetic analysis when these maps are aligned to the corresponding sequenced genome.

The first DNA marker-base linkage map of the common bean, *P. vulgaris* L., was constructed with RFLP markers [14]. Since then, several maps have been constructed using AFLPs [15], RAPDs [16], SSRs [17], and combinations of markers [18, 19]. The conversion of some RFLP markers into STSs [20] opened the possibility of aligning the existing linkage maps with the physical map of the common bean. This manuscript describes the construction of a linkage map of the common bean through the implementation of the genotyping-by-sequencing (GBS) method [3] using a recombinant inbred family derived from two parental genotypes of Andean and Mesoamerican origin with contrasting phenotypes. The nature of this map provides an intrinsic bridge between linkage and physical maps, and presents a functional localization of pCENRs and a landscape of recombinational activity.

## Materials and Methods

### Segregating Progeny

A recombinant inbred family was generated from a cross between Jamapa and Calima, a Mesoamerican and an Andean bean cultivar. Jamapa is a small and black seeded (*c*) [21] bean with an indeterminate growth habit (*Fin*) [22], pigmented hypocotyl (*Hpg*), purple flowers (*V*) [23] with pigmented stripes on the back of the standard, and pigmented calix and floral bracts. Calima is a large seeded mottled bean (*C*), with a determinate (*fin*) growth habit, white flower (*v*) and lacking pigmentation of the hypocotyl (*hpg*). The RI family was propagated by single seed descent and in bulk afterwards to the F_11:14_ generation. A total of 188 recombinant inbred (RI) lines were genotyped in this project. A subset of previously mapped BNG (BeaN Genomic random sequences) markers [14] were also mapped in a subgroup of this RI family as RFLPs and most of the remainder were mapped as PCR markers using high resolution melting (see below). The sequences of the BNG markers have been submitted to GenBank (KM061153—KM061375).

### DNA Extraction

Nuclear DNA for genomic sequencing was extracted from individual RI lines along with the parental genotypes as described elsewhere [24] with two modifications. The first included the use of 96-well cartridges with 1.2 ml tubes, each containing a stainless steel ball (3.98 mm diameter; NationSkander California Corp.). A very young leaf (≤ 2 cm) was collected into each tube, and frozen in liquid nitrogen and stored at -80°C. Later, the 96-well cartridges were placed in liquid nitrogen to freeze the samples before they were ground into a powder in a reciprocal shaker (HT Homogenizer) at 1400 rpm for 40 seconds. The second modification was the inclusion of Triton X-100 in the sample resuspension buffer (SRB). This detergent dissolves plastid and mitochondrial membranes resulting in the release of cytoplasmic DNA to the buffer solution, decreasing cytoplasmic DNA contamination in the nuclear fraction. Immediately after grinding, the powder was resuspended in 800 μl of SRB containing 100 mM Tris.HCl (pH 8), 50 mM NaCl, 0.5% Triton X-100, and 1% β-mercaptoethanol. The 96-well cartridges were spun at 200 g_max_ for 10 min, and the supernatant was discarded afterwards. The pellet was resuspended in 200 μl of nuclear resuspension buffer containing 100 mM Tris.HCl (pH 8), 50 mM NaCl and 50 μg/ml of cloned RNase A, and incubated at room temperature for 15 min. An equal volume of 2X lysis buffer (200 μl) was added to the samples, and after thorough mixing the lysate was incubated at 65°C for 60 min with intermittent gentle mixing. Samples were extracted with 400 μl of chloroform, precipitated with 250 μl isopropanol, washed with 300 μl of Ethanol, air-dried and the DNA pellets were dissolved in 100 μl of 0.1X TE buffer. DNA concentrations were determined fluorimetrically with the Hoescht reagent.

### High Resolution Melting Curve Genotyping

*Glycine max* (soybean) intron-based SNP markers, known to be polymorphic between the parents, were tested across the RILs using high resolution melting (HRM) genotyping. PCR amplification was performed in a 10 μl volume with 6.0 ng of DNA, 200 μM of each dNTP, 1.5 mM MgCl_2_, 100 µM of each forward and reverse primer, 20 µM SYTO 82 [25], along with 0.25 units of Taq DNA polymerase. PCR amplifications were carried out in a LightCycler 480 thermocycler (Roche) programmed as follows: 1 cycle of 2 min at 95°C 55 cycles of 95°C for 30 sec, 58°C for 30 sec, and 72°C for 30 sec; and a final extension step at 72°C for 10 min. Amplified samples were subsequently melted and changes in the melting curves were recorded utilizing the melting curve analysis mode with the following settings: start temperature 68°C, end temperature 90°C, and 32 acquisition points per second in continuous mode with a ramping rate of 0.02°C per second.

### Genotyping-By-Sequencing

*Pst*I GBS libraries were prepared as described by Elshire *et al*. [3] with a few modifications. First, 200 ng of DNA were digested with 20 units of *Pst*I, and 20% of the ligation reaction volume (50 µl) was used for PCR amplification in a 50 µl reaction. In contrast to the original method, each genotype in the population was amplified independently using a two-step PCR protocol (95°C for 2 min; 24 cycles of 95°C for 20 sec and 72°C for 20 sec; 72°C for 5 min; 4°C). The number of amplification cycles was selected to maximize yield and avoid amplification bias towards small amplicons. DNA samples were cleaned each time after ligation and amplification using MultiScreen_HTS_ PCR filter plates (Millipore, MSNU03010). Briefly, 150 µl of water was added to each sample, the diluted sample was added to an individual well in the filter plate, and vacuum filtration was carried out under 10 to 12 inches of Hg tension. After the filter dried out, DNA samples were resuspended in 50 µl of water. Samples were transferred to clean 96 well plate after vortexing the filter plate in an orbital shaker for 30 min at 250 rpm at room temperature. The DNA concentrations of the clean amplicons were measured fluorimetrically with PicoGreen (Life Technologies) and concentrations were confirmed by agarose gel electrophoresis. Two plate pools were constructed using equal amounts of DNA from each well in the plate, and each plate contained the two parents and 94 RI lines. The pooled DNA libraries were size fractionated by electrophoresis in 2.5% agarose (Certified PCR Agarose; Bio Rad, Hercules, CA) gels. Agarose blocks containing DNA fragments in the range of 200 to 500 bp were cut out of the gel, chopped into small pieces, and transferred into Spin-X Centrifuge Tube Filters (Costar, Corning Inc.). These filters were fitted into 1.5 ml microcentrifuge tubes and the DNA was eluted out at 2000 rpm for 5 min. Eluted DNA was cleaned using QIAquick PCR Purification Kit (Qiagen). Purified DNA libraries (S1 Fig.) were quantified and submitted for sequencing in the Illumina HiSeq platform.

### Sequence Data Processing and SNP Calling

**Figure 1:**
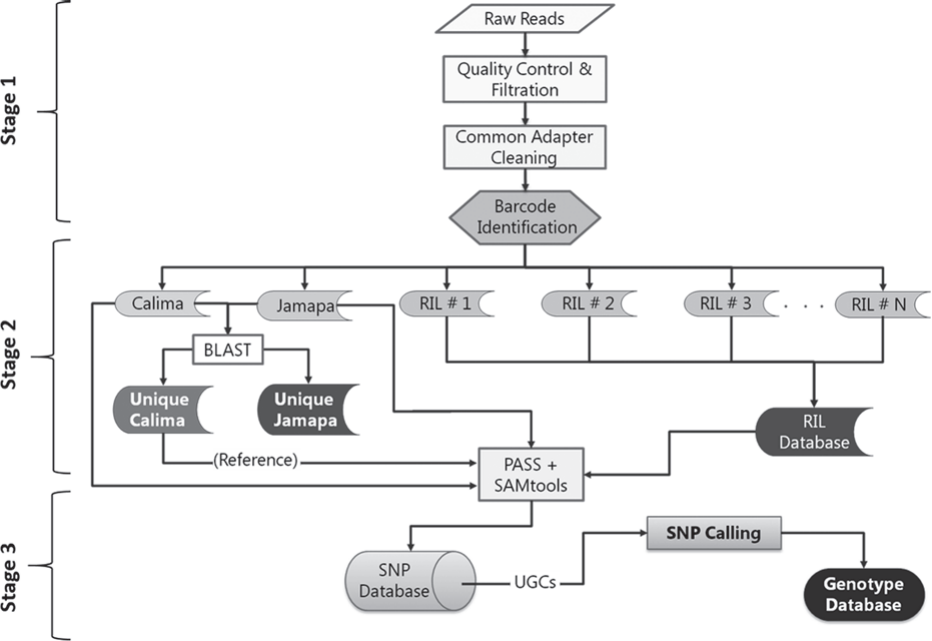
Flow chart illustrating bioinformatics steps used to generate the genotype database for the parental (Calima and Jamapa) and RI lines. Raw sequence data obtained through genotyping-by-sequencing approach was processed in three stages. Stage 1 represent filtration steps and quality check of raw data. Stage 2 depicts separation of sequenced reads using barcode and generation of reference reads set. Stage 3 represent the SNP calling and genotype database generation steps.

Sequence data were processed in stages using multiple bioinformatics tools, and without a whole-genome reference sequence (Fig. 1). The processing pipeline was divided into three major stages. In stage 1, bases with low quality scores (<20) and common adapter sequences at the 3’end, if present, were trimmed off. In addition, reads without an exact barcode at the 5’ end were removed from the stack.

In the second processing stage, the cleaned and filtered reads were separated out into their respective parent or RIL-specific files according to the exact barcode sequence detected at the 5’end. Given the sequencing approach, most reads within a RIL had duplicates. These were removed from the reference (parental) genotypes after identifying them with NCBI BLAST+ [26], PASS v2.1 [27], and SAMtools-0.1.19 [28], allowing the generation of a unique set of reads for each parent from the combined set obtained from both libraries: C-Ref and J-Ref for Calima and Jamapa, respectively. Reads with a redundancy below four were excluded to reduce the probability of including sequencing errors in the analysis. The C-Ref (J-Ref) was assembled by concatenating the unique sequences which were joined by a string of 200 Ns (S2 Fig.); thus, each base in the C-Ref (J-Ref) was assigned a unique coordinate for downstream analysis.

The final processing stage dealt with SNP identification and assembly of the RI family genotype database. The VarScan 2.3.5 [29] program analyzed RIL files (SAM) generated by PASS v2.1 against C-Ref for base calling at each position and identification of SNPs. This analysis identified sequences missing in the Jamapa file, but their presence in the majority (>85%) of the RIL family indicated that these apparent deletions were due to a sampling problem. A second alignment was carried out using the J-Ref file as reference to identify sequences that were present in J-Ref and absent in Calima; these alignments also showed that the same type of sampling error had occurred in the other parent. No indels were included in the linkage analysis. As our population was comprised of RILs (at least 11 generations of selfing), we used only homozygous base positions for SNP calling. Accordingly, a nucleotide position was called homozygous when an observed base frequency was greater than 0.95. Subsequently, a SNP database file consisting of a matrix of base calls from each genotype corresponding to the reference sequence was constructed using a Python script. Comparing the parental base calls in the SNP database file allowed identification of polymorphic base positions. Positions where a deletion was detected in the non-reference parent were examined by searching the RIL database at that position to determine whether the putative deletion was segregating in the RI population. If the sequence was present in the population, then the deletion in the non-reference parent was considered a sequencing artifact. If no base displayed a polymorphism, it was ignored, but if the RI family showed segregation, then it was considered as a real SNP and it was added to the database. The same procedure was repeated for apparent deletions in the reference parental genome. Allelic sequences used in the construction of the linkage map are available at http://dx.doi.org/10.6084/m9.figshare.1243359.

### Linkage Analysis

Segregation and linkage analyses were carried out with the GBS-derived SNP markers (DiM) that satisfied all quality control standards and passed all the filtering processes, along with the six *Glycine max* intron-based SNP markers (unpublished data), the BNG markers previously mapped as RFLPs [14], and nine phenotypic markers. Chi square tests were carried out to detect the presence of segregation distortions in the analyzed markers.

MadMapper [30] along with RECORD (REcombination Counting and ORDering) [31], and MapMaker 3.0 [32] (version modified by Russell Malmberg, http://www.plantbio.uga.edu/007Erussell/) were used to perform the linkage analysis and map construction. An initial analysis was conducted with MadMapper in which the markers were grouped using MadMapper_RECBIT script with a recombination cutoff value set to 0.2, bit score cutoff to 100, allele distortion to 0.33, and with the NOTRIO option ON. Marker order on each identified linkage group was determined by running the MadMapper_XDELTA script. The order was also verified by running the RECORD software with maximum additional recombination value set to default, with 200 iterations, the Kosambi map function, and gap size set to 20 cM.

The second map was constructed using MapMaker 3.0 using the same marker set used earlier with MadMapper. Markers were first assorted with the GROUP command with default linkage criteria of LOD 10 and 25 Kosambi cM. The markers in each group were ordered using an informativeness criteria set at 2 cM and a minimum of 170 informative RI lines, and a multipoint criterion set at an LOD threshold of 4.0, a window size of 7 and a strict LOD of 5.0. Markers excluded under the previous criteria were assigned a position by the TRY or PLACE commands using an LOD exclusion threshold of 3.0. Linkage groups named A to K in previous maps [14] were renamed according to chromosome numbers assigned to these groups in the newly released genome sequence [33]. The CheckMatrix [30] program was also utilized to graphically validate the marker order on each linkage map generated to address the potential error caused by missing values or genotyping errors.

### Marker Distribution Analysis

The Poisson distribution was used to test the distribution pattern of markers in the genome. If markers are randomly distributed in the genome, then markers at a given interval would closely follow a Poisson distribution pattern [34, 35, 36]. The test was carried out by dividing the linkage groups into a fixed interval length. This analysis was carried out independently for six different intervals lengths (n = 1, 2.5, 5, 10, 20, and 40 cM). The distribution pattern of the markers in each interval was evaluated by calculating the probability of x markers falling within a given interval. This probability was calculated with the probability mass function of the Poisson distribution,

$$p(x,\lambda )=\frac{e^{-\lambda }\lambda^{x}}{x!},x=0,1,2,...$$

where λ is the mean number of markers per interval. Significant differences between observed and expected frequencies of the number of markers per interval were detected through Chi square tests.

### Relationship between Linkage and Physical Maps

Alignment of the linkage map to the physical map was performed with the BLAST+ algorithm through a BLASTn search for all the mapped sequences against the *P. vulgaris* genome sequence obtained from www.phytozome.net [33]. The mapped sequences comprised all BNG markers and the unique Calima allele reads containing the informative SNP. The best match of reads with multiple BLAST hits was used to infer the map position. The actual position of SNP on the reference genome was located using a custom Python script while taking sequence orientation into consideration. *P. vulgaris* and *G. max* EST collections found in the NCBI databases along with Soybean coding sequences [37] were utilized to search for coding sequences across common bean chromosomes.

### Genomic Sequencing

Whole nuclear DNA from the parental lines were sequenced using the SOLiD sequencing platform in 2010. This platform generated 197,948,596 high quality reads for Jamapa and 181,367,575 for Calima. These 50 bp reads produced an average sequencing depth of approximately 15.6X and 14.3X for Jamapa and Calima, respectively. The SOLiD reads were utilized to generate parental alignment against the available reference genome assembly, and BNG marker sequences. These alignments allowed genome-wide identification of SNPs between parents, along with the distribution of the *Pst*I cut sites.

## Results

### Sequencing Library

*Ape*KI, the methylation sensitive enzyme used by Elshire *et al*. [3], was found to cut bean DNA at a relatively high frequency and was deemed unsuitable for GBS (S3 Fig.). In contrast, *Pst*I is a methylation sensitive restriction enzyme that cuts bean gDNA very infrequently (S3 Fig.); it had been used earlier to generate a library of low copy random clones suitable for RFLP detection and linkage map construction [38]. For this reason, *Pst*I was chosen to construct the GBS sequencing libraries.

The DNA preparations were found to contain template that was responsible for the disproportionate amplification of a small amplicon (∼90bp) with the Illumina primers. However, this amplicon was absent when the procedure was carried out with Lambda DNA, or DNA from purified nuclei or chloroplast DNA (provided by Dr. K Cline, University of Florida), but it was present when mitochondria DNA (provided by Dr. C.D. Chase, University of Florida) (S4 Fig.) was used as template. The small amplicon problem was minimized in two ways. First, Triton X-100 was added to the sample resuspension buffer used in the first step of DNA extraction to dissolve organelle membranes and release their DNA into the supernatant. Second, the PCR protocol was changed to a 2-step protocol alternating between a denaturation step at 95°C and an annealing/extension step at 72°C. The success in eliminating the artifactual amplicon suggested that annealing to the organelle DNA was only partial and unstable. Amplification, cleaning and quantitation of individual lines helped ensure equal representation of each line in the pool destined for sequencing. Furthermore, gel extraction of the pooled amplicons allowed selection of a desired range of DNA fragments, and also provided an additional control step to remove any potentially interfering small molecular weight sequences which could have reduced sequencing quality and efficiency of target fragments.

### Processing Reads and SNP Calling

The Illumina HiSeq platform yielded 245.6 million reads (101bp) from the first library (Plate-1, Lane 1), while the second library (Plate-2, Lane 2) generated 260.8 million reads. Following quality control and filtration (Fig. 1, Stage 1), the number of reads in the first library was reduced to 161 million (34.5% reduction), while the second library was left with 154.1 million reads (40.9% reduction) (S5 Fig.). On average, each genotype yielded 1.6 million useable reads, with a range between 0.2 and 4 million (S6, S7 and S8 Figs.). The sequence count for Calima was 1,812,118 from the first library and 1,868,944 from the second, while the number of reads from Jamapa was 1,761,153 and 1,414,062 from the first and the second library (S6 Fig.), respectively. The second processing stage identified a total of 75,867 unique Jamapa reads and 78,966 unique Calima reads, representing 1.20% and 1.24% of the 637 MB genome [39], respectively. A total of 2,264 SNPs were identified between the parents, with an average read depth of 42x (Jamapa) and 49x (Calima). As expected, the majority of the SNPs represented transition events (55.74%) as compared to transversion events, with the most common allele variant (28.3%) resulting from a C↔T change.

For the purpose of identifying informative polymorphisms for the construction of a linkage map, further selection was carried out to only include SNPs that were present in at least 85% of the RI lines. Such stringent selection reduced the number of highly informative SNPs to 1,473, a 35% reduction. These SNPs were detected in a total of 1,112 reads; for this reason, the SNP with highest quality score was selected in reads with multiple SNPs.

### Linkage Map

**Figure 2:**
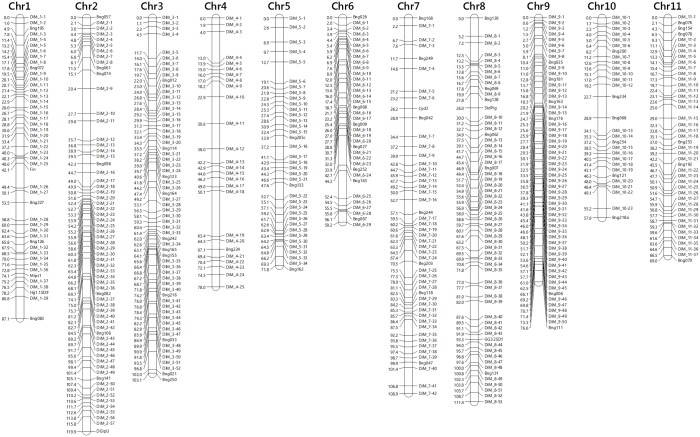
Linkage map (LOD 3.0) of *Phaseolus vulgaris* L. with 513 unique marker loci distributed among the 11 chromosomes with an average inter locus distance of 1.84 cM.

Linkage analysis was carried out with a total of 1,341 marker loci, which included 1,112 SNP markers, 214 RFLP-based markers, six soybean-derived SNP markers, and nine phenotypic markers. Both MadMapper and MapMaker 3.0 sorted the markers into 11 linkage groups, a number that matches the basic chromosome (Chr) number for the species (2n = 2x = 22, n = 11). FISH analysis with BNG markers was used previously to demonstrate that each linkage group corresponds to a distinct chromosome [40]. MadMapper generated a map with 987 markers distributed over 1,181cM; 271 markers were not included because they shared the same recombination pattern with at least one other marker included in the map. In contrast, MapMaker 3.0 generated a map with 513 unique loci covering 943 cM. The JOIN HAPLOTYPE command of MapMaker 3.0 identified and removed from the analysis 739 markers that belonged to recombinationally indistinguishable groups of markers while selecting the most informative one from each group to be included in the map. An additional set of 89 markers was not included in the map because they could not be assigned a position on the map with an LOD threshold of 3.00. Inserting these markers into the map increases both the total map distance and the chances of placing them at the wrong position. The final MapMaker 3.0 linkage map includes 442 SNP loci (DiM), 66 RFLP-based markers, three soybean-derived SNP markers, and two phenotypic marker loci (Fig. 2, S1 and S2 Table). Although both mapping packages generated similar linear marker orders for each linkage group, alignment of these maps to the physical map identified the MapMaker 3.0 map as the most accurate (see section below), and for this reason it was selected for further analysis.

The relative position of each marker in their respective linkage group was verified by two methods. First, no linear order errors were detected after testing the results with the RIPPLE command using a LOD threshold of 3.0 and a window of five marker loci. In addition, the CheckMatrix software produced a visual (heat map) representation of the linkage relationships a marker has with all the other markers from the entire linkage map. CheckMatrix utilizes red to denote strong positive linkage and blue for no linkage. A robust linkage map produces a strong red diagonal (top-left to bottom-right) indicating a strong linkage of a particular marker to its neighboring markers on its representative linkage group. The heat map (S9 Fig.) generated by 513 markers included in the MapMaker linkage map validated the linkage groups and linear arrangement. Furthermore, test results with the Poisson distribution using different interval lengths (1.0, 2.5, 5, 10, 20, 40 cM) clearly showed that the markers are not randomly distributed in the genome (P < 0.001; S10 Fig. and S3 Table). Finally, alignment with the genome sequence also showed excellent agreement with the mapped linear order (see next section).

**Figure 3:**
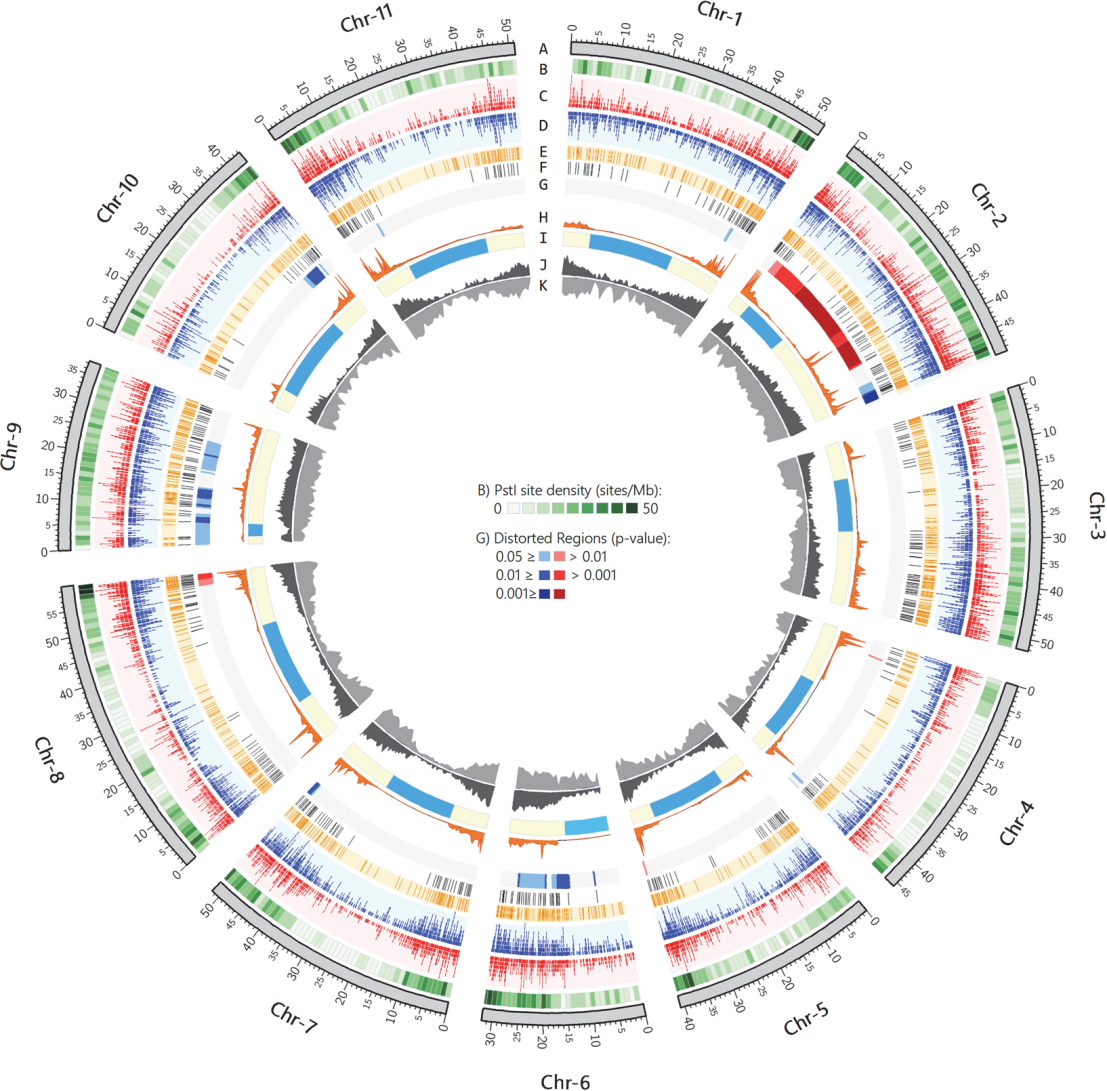
Circos plot depicting the distribution of various genomic attributes along the physical map of the 11 *P. vulgaris* chromosomes. (A) Physical chromosome (Mb) of common bean. (B) Genome wide heat plot of potential *Pst*I sites detected by *in-situ* analysis. (C, D) Frequency distribution of Jamapa (blue) and Calima (red) reads obtained through GBS, (y-axis: range 0–160 reads/Mb). (E) Position of GBS-based SNPs identified between parental lines. (F) Physical location of 513 recombinationally unique markers utilized to construct the linkage map. (G) Heat plots highlighting regions with transmission ratio distortions in favor of Jamapa (Blue) or Calima (Red) alleles. (H) Density plot of recombination rate (y-axis: 0–35 cM/Mb). (I) Estimated pericentromeric region (light blue). (J) Density plot of coding sequences (CS) along chromosomes (y-axis: 0–178 CS). (K) Frequency distribution of genome-wide *in-situ* identified SNPs between Jamapa and Calima (y-axis: 0–2000 SNPs).

The linkage groups varied in length from 58 cM for Chr 10 to 120 cM for Chr 2 (Table 1). Likewise, the groups also varied in marker content from 38 for Chr 4 to 140 for Chr 2, and the average inter-marker distance ranged from 1.39 cM for Chr 9 to the highest at 3.00 cM for Chr 4, while the overall inter-marker average distance was 1.84 cM. The biggest gap of 13.3 cM was observed on Chr 4. The uneven marker distribution and variable marker density along the genome are in good agreement with the results of the Poisson distribution tests. Segregation analysis along the linkage groups uncovered sectors with significant deviations from the expected Mendelian ratio of 1:1. These segmental distortions were detected in all chromosomes with the exception of Chr 3, with Chr 2 showing the longest distorted sector (∼90 cM) including 43 markers (Fig. 3G). The linkage analysis also detected 55 markers with at least one double cross over event.

**Table 1:** Distribution of markers on individual linkage groups.

**Chrom^*a*^**	**Lnk. Group^*b*^**	**cM**	**Total markers**	**Total unique loci**	**Avg. inter loci distance (cM)**
1	H	87.1	94	47	1.85
2	D	119.9	140	65	1.84
3	C	103.1	120	63	1.64
4	B	78.0	38	26	3.00
5	E	71.8	85	37	1.94
6	G	59.2	74	36	1.64
7	A	108.9	78	50	2.18
8	F	111.4	130	61	1.83
9	K	76.6	102	55	1.39
10	I	57.9	47	29	2.00
11	J	69.0	79	43	1.60
		**942.9**	**987**	**512**	**1.84**

^*a*^ Reference chromosome

^*b*^ Linkage group [14]

### Linkage Map vs. Physical Map

**Figure 4:**
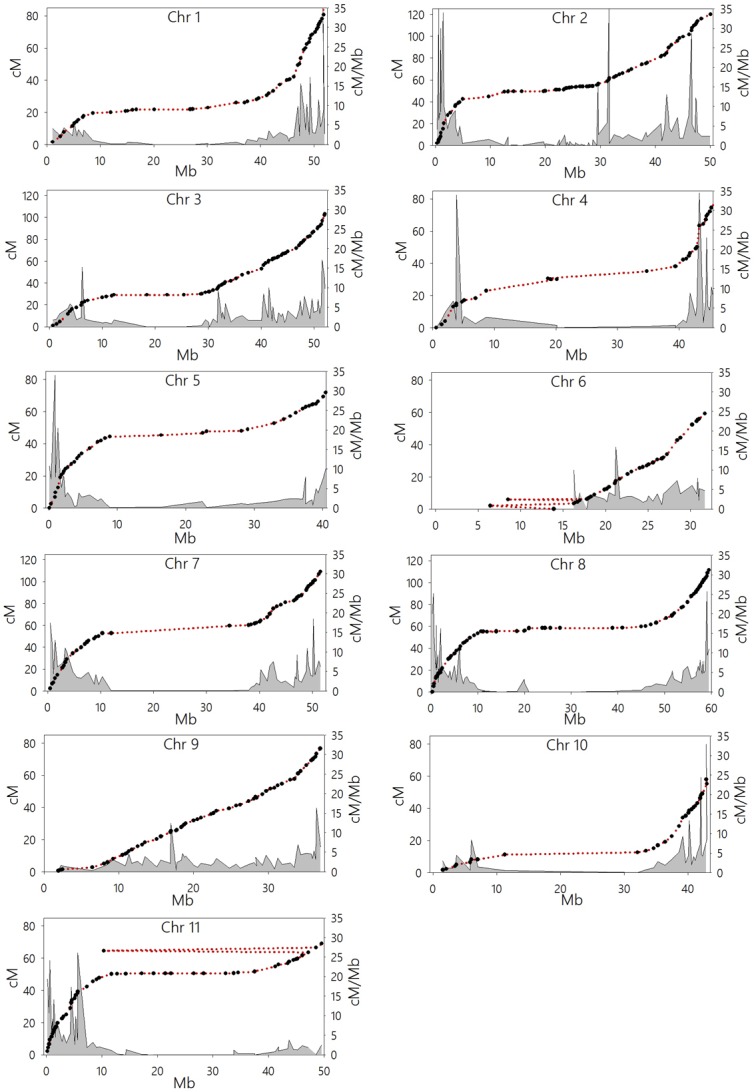
Scatter plot displaying the relationship between centiMorgan distances (left Y-axis) and physical distances (X-axis, Mb) along the 11 chromosomes of *P. vulgaris* L. Black circles represent mapped markers (513+739). Red dotted line connects neighboring markers for visual ease. First order differential plot (grey density plot) represent cross-over rate across a given chromosome (right Y-axis, cM/Mb).

Aligning the MapMaker linkage maps to the physical map revealed four discrepancies (Fig. 4). Markers DiM_6–1 and DiM_6–6 were mapped ∼6Mb and ∼9Mb away from their sequenced positions on chromosome 6 (linkage group G), while the marker DiM_11–36 on Chr 11 (linkage group J) was mapped ∼37Mb away from its current position in the genome sequence. BLAST searches against *P. vulgaris* and *G. max* whole genomic sequences indicated that these three marker sequences belonged to single copy coding regions. Also, marker Bng164 was mapped to Chr 3, but has been assembled on Chr 4 of the reference genome instead. The alignment also identified three mapped marker sequences which were not detected in the reference genome after a BLAST search (DiM_3–25, DiM_4–19 and DiM_10–20).

Scatter plots were used to analyze the relationship between linkage map distances (y-axis, cM) and physical distances (x-axis, Mbp) along each chromosome (Fig. 4). These plots revealed that the linkage map covers 95.83% of the sequenced genome, varying from 97.0% to 99.9% for 10 of the 11 chromosomes. Only 55.6% coverage was detected for Chr 6. Joining contiguous points in the scatter plots produced curves in which the slope is proportional to the recombinational activity. Accordingly, examination of these curves revealed the distribution pattern of recombination activity along each chromosome. Ten of the 11 chromosomes displayed similar patterns in which a middle segment displaying a gentle slope was flanked by steep slope segments. The shallow section of these plots is taken to represent the pCENR of each chromosome. In contrast to the other chromosomes, Chr 6 displayed a single one-sided segment with a steep slope.

To examine in greater detail the distribution of CO along the chromosomes, we calculated the first derivate of map distances relative to physical distances (dcM/dMb) (Fig. 4); the area under the derivative function was shaded in grey. This procedure clearly identified sectors in which the first derivative function is so close to zero (< 1 cM/Mb) that the grey shading is not apparent. These sectors provide a functional identification of pCENR, which have extremely low marker density, suppressed recombination, and variable lengths. The smallest pCENR region was detected in Chr 9 with an estimated size of 6.06 Mb representing 16.4% of the chromosome, and the largest one was detected in Chr 1 extending over 31 Mb representing about 51% of the entire chromosome. Excluding Chr 6, pCENR represent approximately 45.6% of the sequenced genome (Table 2).

**Table 2:** Summarized comparison between linkage and physical maps of each chromosome.

			**Recombinational *p*-arm**			**Pericentromeric Region**				**Recombinational *q*-arm**		
**Chrom**	**Total cM**	**Total Mb**	**cM**	**Mb**	**Avg. cM/Mb**	**cM**	**Mb**	**% of chrom**	**Avg. cM/Mb**	**cM**	**Mb**	**Avg. cM/Mb**
1	87.1	52.2	19.5	7.9	2.5	6.5	26.8	51.3	0.2	61.1	16.8	3.6
2	119.9	49.9	42.3	4.6	9.2	7.6	15.1	30.3	0.5	70.0	30.0	2.3
3	103.1	52.3	28.4	11.7	2.4	1.5	16.8	32.1	0.1	73.2	23.7	3.1
4	78.0	45.9	30.6	6.6	4.6	7.4	20.6	44.9	0.4	40.0	18.1	2.2
5	71.8	40.8	19.1	7.9	2.4	8.4	24	58.8	0.4	44.3	8.8	5.0
6	59.2	31.9	NA	NA	NA	NA	NA	NA	NA	NA	NA	NA
7	108.9	51.7	57.1	11.4	5.0	2.4	22.4	43.3	0.1	49.4	17.4	2.8
8	111.4	59.6	55.9	14.0	4.0	2.7	27	45.3	0.1	52.8	18.6	2.8
9	76.6	37.4	NA	0.4	NA	2.7	6.0	16.4	0.5	73.9	30.5	2.4
10	57.9	43.2	8.2	5.0	1.6	4.4	26.1	60.4	0.2	45.3	11.1	4.1
11	69.0	51.5	50.1	11.7	4.3	0.8	25.8	50.1	0.0	18.1	12.9	1.4

“NA”: Data not available

Our data set did not allow us to identify the relative location of the pericentromeric region of chromosome 6, and it suggested the chromosome might be telocentric (Fig. 4). However, this chromosome, known as linkage group G, was characterized previously in Calima as acrocentric by Pedrosa *et al*. [40] using fluorescent in situ hybridization. The *p* arm of this chromosome is dominated by a block of 45s rDNA repeats, while the pericentromeric region of the *q* arm is occupied by a block of 5s rDNA repeats. In summary, the absence of SNP markers on the *p* arm made it impossible to define the pericentromeric region of Chr 6.

The average recombination rate along chromosome arms outside the pCENRs fluctuates between 2.8 to 5.7 cM/Mb. However, an examination of the first derivative profiles reveals a punctuated distribution of recombination, which can be seen as peaks representing “hot spots” of recombination reaching up to 34 cM/Mb. These peaks correspond to apparent gaps in the linkage map (Fig. 2), particularly noticeable in chromosomes 1, 2, 4, 5, 8, 10, and 11. The distribution of these “hot spots” is not uniform along or between chromosomes. For instance, Chr 2 has four contiguous peaks on the *p* arm and three on the *q* arm, while chromosome 4 has a single peak on the *p* arm and a couple of minor peaks on the *q* arm. In contrast, Chr 3, 6, and 9 have one or two medium size peaks (∼15 cM/Mb).

## Discussion

We have successfully implemented the genotyping-by-sequencing methodology [3] to construct a high-density linkage map of the common bean (*P. vulgaris*). The map was constructed with a RI family (F_11:14_, n = 188) derived from a cross between an Andean and a Mesoamerican accession. GBS is a very effective and efficient map construction method as compared to hybridization or PCR-based methodologies, which are labor-intensive, time-consuming and costly [14, 17, 41].

Two modifications were introduced to reduce and eliminate the dominant low molecular weight amplification product, which was associated with the presence of mitochondria DNA. First, a mild detergent was included in sample resuspension buffer to dissolve and release mtDNA into the first supernatant. Subsequently, PCR amplification was converted into a 2-step amplification in which annealing and extension were carried out at 72°C. The high annealing temperature interfered with spurious primer annealing to contaminating mtDNA.

One of the challenges of GBS is obtaining an optimum balance between a reduction in genome representation, and an increase in multiplexing and sequencing depth, all along minimizing the extent of missing data points, a problem that has been documented in the literature [42]. We have introduced two modifications in the procedure to reduce the number of missing data. First, instead of a PCR amplification of the pool of ligated samples, we PCR amplified each sample individually, cleaned them, quantitated them, and used this information to construct the pool with equal contribution of the different genotypes. Post-amplification pooling prevents the dominance of a few genotypes during the amplification of the pool. The second modification was the size fractionation of the pooled DNA libraries using agarose gel electrophoresis. This step ensures the elimination of unincorporated primers which would compete with the DNA sample for binding sites on the Illumina sequencing plate, exclude large DNA fragments (> 500 bp) which generate low density/contaminated amplification clusters, and overall uniformly reduces the complexity of the sample. An approximate average of 158 million high quality reads per 96 genotype library per lane were obtained from the HiSeq 2000 platform. Thus, on average we obtained about 1.64 million reads per genotype with a standard deviation of 0.6 million reads. A few outliers were detected with a read count as low as 0.2 million and as high as 4 million. These extremes could be due to random chance during preparation of the sequencing lane, or due to low quality DNA resulting in a bias during library construction.

Although we identified 2,264 polymorphic sites between the parental genotypes, the total number of SNP markers was reduced for two reasons. First, only two thirds were present in at least 85% of the RI family. Second, among the remainder SNPs, several were detected within a single 100-base read and therefore only one of those had real informative value for mapping. This problem can be further reduced by either increasing the sequencing depth through reduction of the pool size, narrowing the fragment size range targeted for sequencing, and/or increasing the number of sequencing lanes. A practical solution to the missing data problem once it has occurred is imputation, a computational approach that uses probabilistic models to estimate genotypes [43, 44]. However, extreme caution must be exercised when implementing this approach.

The GBS-derived SNP-based linkage map of common bean was constructed via an ad hoc bioinformatics pipeline without the assistance of a reference genome because the sequence of the bean genome was released subsequent to our research [33]. The availability of a reference genome can greatly facilitate processing of GBS data and also imputation of missing data points [43, 44]. However, one must be aware of the potential problems when using a reference genome as it may carry deletions with respect to the experimental material, minor or major chromosome rearrangements, or contain assembly errors. For instance, we detected three sequences (DiM_3–25, DiM_4–19 and DiM_10–20) that appeared to be deleted in the reference genome G19833. Deletion of these sequences is supported by their absence in SOLiD sequencing reads of the reference genome obtained at UF (G19833, 15X coverage), but not in SOLiD reads of the wild accession G23419, or the parental genotypes. In addition, we mapped marker Bng164 to Chr 3, but this sequence was assembled in Chr 4 of the reference genome. This is most likely a misassembly because this 1,386 bp *Pst*I random genomic clone has been previously mapped to Chr 3 (Linkage Group C) in a different population (Vallejos *et al*. 1992), and a chromosome section delimited by markers flanking Bng164 is syntenic with a section of Chr 17 in *G. max*, but no synteny can be detected between any sections of Chr 4 of *P. vulgaris* with Chr 17 of *G. max*. Furthermore, no spurious map positions were detected with CheckMatrix analysis (S9 Fig.). Thus, deletions and misassemblies can create problems when using GBS data for genome-wide association studies. The procedures outlined here for processing GBS data can be easily applied to species currently lacking a reference genome sequence.

We have constructed a 513 marker linkage map that extends over 943 cM (Kosambi function) with a LOD threshold of 3.0. Out of the 1,341 markers analyzed, 89 were left out of the map because they could not be positioned unequivocally with an LOD of 3, and 739 were not included in the analysis because they were recombinationally indistinguishable from one or more markers in a group. This phenomenon could be ascribed to either the lack of true recombinants given the size and type of the mapping population, or to the fact that some markers had missing data points that prevented the proper identification of recombinants. Nevertheless, the map presented here has an average map density of one marker every 1.84 cM, which will increase the resolution and precision of QTL mapping [45].

Statistically significant transmission ratio distortions (TRD) favoring one of the two alleles were detected in all chromosomes, with the exception of Chr 3. The major distortions were observed in Chr 2 with an excess of Calima alleles at one end and Jamapa alleles at the other end. Observed TRDs could be explained by random sampling error or by biological phenomena like pre- and post-syngamic selection. Given the extent of diversity between the gene pools [46], it is not surprising to detect TRD in this inter gene pool cross as such distortions have been found to be correlated with genetic diversity [47]. TRDs can also be explained by gene conversion phenomena in which one of the homologs is preferentially targeted for DSB. This explanation is supported at least in part by the observation that several of the TRDs we report here coincide with hot spots of recombination (Fig. 3G and H). Future characterization of the mechanism controlling TRDs will facilitate gene transfer between gene pools and perhaps reveal incipient mechanisms of speciation in the common bean.

The recent availability of the *P. vulgaris* genome sequence [33] has made it possible to examine several features of the recombinational landscape of the common bean, including the origin and characteristics of the markers used in the construction of the linkage map. For instance, in spite of their extreme biased distribution outside the pCENR (55% of the sequenced genome), the *Pst*I-linked markers (GBS-SNPs and BNGs) provided complete genome coverage and displayed a mostly uniform distribution in the linkage map. To address this apparent incongruence, we examined the genomic distribution of potential *Pst*I sites (Fig. 3B), coding sequences (Fig. 3J), and SNPs (Fig. 3K). We sampled 10 Mb sequences from both inside and outside regions we had identified as pericentromeric. Next, we extracted the sequences (exons and introns, including 5’UTRs and 3’UTRs) from all the genes in the 10Mb samples and aligned SOLiD-platform reads from the two parental genotypes to both the entire genomic DNA and the gene sequences. These alignments showed that the average frequency of SNPs was marginally higher in the genomic sequences than in the gene sequences. Further analysis of the Circos graphs (Fig. 3) showed a high degree of correlation between the distribution of coding sequences and potential *Pst*I sites, with a relatively high frequency outside the pCENR.

Superimposed on the distribution of potential *Pst*I sites is the pattern of plant genomic methylation that targets these sites. *Pst*I is a methylation sensitive restriction enzyme, which will not cut DNA if any of the cytosines in the ‘CTGCAG’ sequence is methylated [48, 49, 50, 51, 52]. In plants, chromomethylase 3 (CMT3) maintains cytosine methylation of CHG sites, and these include *Pst*I recognition sequences [53]. It is also known that CHG methylation occurs at a much higher frequency in the pericentromeric region than outside of it, and that transcribed regions display CHG hypomethylation with respect to their upstream or downstream regions [54]. Taken together, the relative low frequency distribution of potential *Pst*I sites coupled to the high levels of CHG methylation in the pericentromeric region, and the pattern of CHG methylation in and around gene sequences explain in great part the success of using *Pst*I-based markers for complete common bean genome coverage. Furthermore, suppression of recombination in the pericentromeric region adds to the apparent “uniform” distribution of markers in the linkage map. In summary, results obtained with the Poisson distribution test represent and mathematical evaluation of *Pst*I related marker distribution reported here.

A small number of double-CO events were also observed and confirmed. These events are often observed with suspicion in primary progenies due to the known CO interference mechanisms in play during meiosis. A double CO in an advanced RI lines can be due to either separate CO events in close proximity in subsequent generations or due to gene conversion events. Such a double-COs would occur without suppressing CO interference. However, a noninterfering CO mechanism has been detected in yeast [55], mouse [56], tomato [57], and Arabidopsis [58], and could explain some of the double CO events we detected in this progeny. Basu-Roy *et al*. [58] have reported higher proportion of noninterfering COs towards the arms as compared to the central region in the majority of Arabidopsis chromosomes.

Pericentromeric regions have been loosely defined and usually identified cytologically as heterochromatic regions surrounding the centromere, and also by hybridization [59]. Alignment of the linkage map to the genome sequence provided a clear graphical and functional definition of the pericentromeric region. The structure of pCENR appear to evolve at faster rate than that of the rest of the genome, often accumulating more repetitive sequences [60]. In this regard, one might speculate that the smallest pCENR of Chr 9 might be in the initial phase of its evolution or could also be the oldest and most highly evolved pCENR in the genome. The pCENRs represent approximately 45% of the sequenced common bean genome, and as it is the case in most species recombination is severely repressed within this region [5, 61]. The average recombination frequency for the euchromatic arms is approximately 1 cM/285 Kb, but local calculations identified recombination hotspots with rates as high as 1 cM/30Kb. This rate may underestimate the true rate given the relatively low density of markers as opposed to calculations involving haplotype sequence. However, hotspots identified here could be characterized in greater detail by obtaining the genomics sequences of informative recombinants identified in this project. Recombination hotspots are in great part defined by the affinity of hotspot DNA sequences to proteins involved in DSB [62, 63], and have been reported for Mimulus [6] and Arabidopsis [7]. In these species, these hotspots have been detected preferentially at the 5’ of CDS and in exons rather than introns. Comparisons of the position of recombination hotspots between the RIL population analyzed here and hotspots in other populations, including one between a wild and a landrace of beans generated in our lab (unpublished), and the Redhawk x Stampede RIL population [33] revealed that some hotspots are common to different segregating populations and some are cross-specific. Mapping recombinational activities on the physical map provides relevant information that will offer reasonable expectations about the resolution that can be attained in gene mapping projects, particularly QTL mapping, map-based cloning, the expected rate of linkage decay in genome association studies along each chromosome, and in improving algorithms for genotype imputation.

## Conclusion

The high levels of CHG methylation in the pCENR along with hypomethylation of these sites in CDSs layered on top of the relatively high frequency of potential *Pst*I sites outside the pCENR contributed to the successful implementation of GBS [3] for the construction of a high density linkage map of *P. vulgaris*. The map on average had a marker every 1.84 cM, and alignment of this map with the genome sequence revealed a genome coverage of nearly 98%. This alignment also helped with the identification of the pCENRs on each chromosome where recombination is highly suppressed, and with the identification of recombination hotspots. Mapping the recombinational rates along the genome can help geneticists assess the level of resolution, at the molecular level, that can be achieved with mapping projects of traits of different complexities, guide map-based cloning projects and interpret GWAS data. Finally, identification of recombination hotspots on a chromosome can lead to the identification and characterization of the associated DNA sequences and proteins that are part of the responsible mechanism.

## Supporting Information

S1 Fig Electrophoretic distribution pattern of purified DNA fragments from size selected GBS libraries (Lanes 2 and 3). Electrophoretic separations were carried out in a 2% agarose gel stained with SYBR Gold nucleic acid stain. Molecular weight markers: Lambda DNA digested with *PstI* (Lanes 1 and 4).
(TIF)

S2 Fig Concatenation of unique parental reads into a single fasta sequence using a bridge sequence comprised of 120 ‘N’. (TIF)

S3 Fig Restriction pattern of Jamapa DNA digested with *Pst*I (Lane 2) and *Ape*KI (Lane 3). For size reference Lambda DNA digested with *Hin*dIII is shown in lane 1.
(TIF)

S4 Fig Agarose gel (2%) electrophoresis showing differential amplification of mitochondria DNA. (1) *Pst*I cut Lambda DNA (100ng), (2) *Pst*I GBS library of Jamapa using purified nuclear DNA (3) Jamapa GBS library total DNA extractions (nDNA + mtDNA).
(TIF)

S5 Fig Pie charts (A, B) representing the percentage of high quality filtered reads obtained from the two sequenced libraries. Improvement in quality score per base in each library after applying quality control filtration step (C, D).
(TIF)

S6 Fig Bar chart representing the total number of filtered reads obtained for each sequenced genotype. Average parental genotype reads count are represented by J (Jamapa) and C (Calima).
(TIF)

S7 Fig Histogram representing the distribution of recombinant inbred lines according to the number of filtered read counts obtained through sequencing. (TIF)

S8 Fig Histogram representing the distribution of recombinant inbred lines according to the percent low quality reads (non-usable) observed prior to filtration and quality control steps. (TIF)

S9 Fig Heat Map showing degree of linkage between markers spread throughout genome. Strong linkage (red color) was observed between neighboring markers on the same chromosome; while weak/no linkage (green/blue) was observed between markers located on separate chromosomes. Yellow islands chromosomes.
(TIF)

S10 Fig Expected and observed marker frequency in 5cM interval of the linkage map. Observed data did not follow a Poisson distribution pattern as the expected data thereby indicating non-random distribution of markers in the intervals.
(TIF)

S1 Table Brief specifications of mapped markers. (DOCX)

S2 Table Primer sequences corresponding to mapped soybean-derived SNP markers. (DOCX)

S3 Table Chi-square test to detect randomness in marker distribution within defined intervals across the linkage map. (DOCX)
