# Supplementary figures and images for: Punctuated Distribution of Recombination Hotspots and Demarcation of Pericentromeric Regions in Phaseolus vulgaris L
Source: PLoS One. 2015 Jan 28;10(1):e0116822. doi: 10.1371/journal.pone.0116822 (PMC4309454; doi:10.1371/journal.pone.0116822)

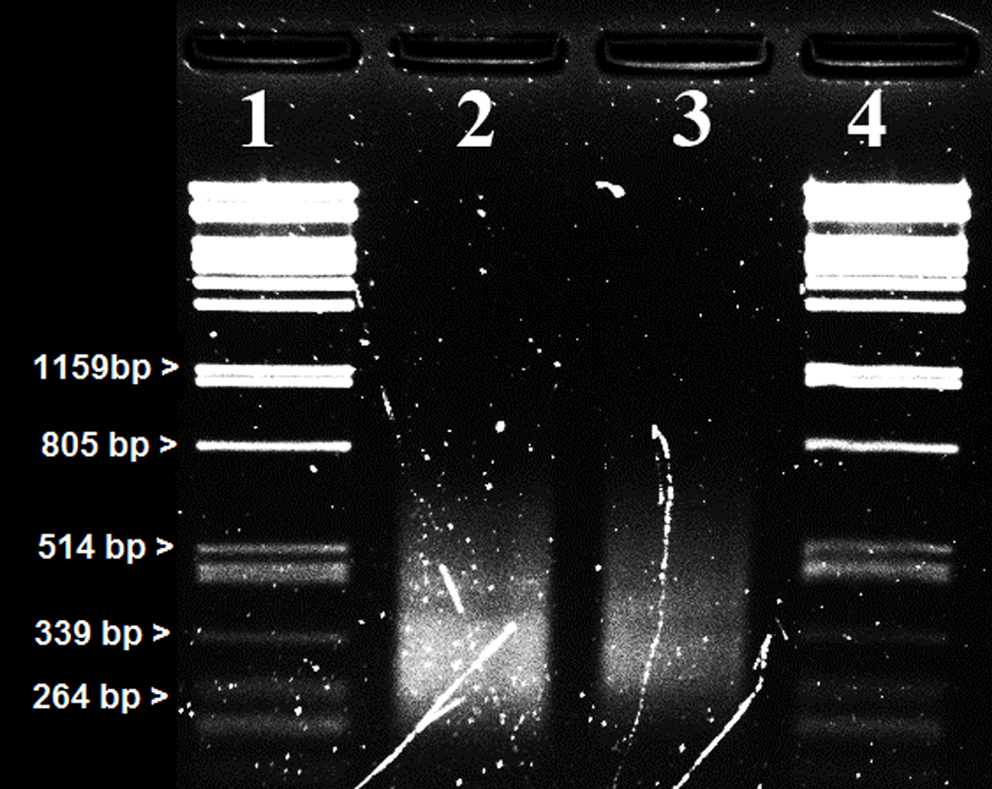

Supplement: S1 Fig — Electrophoretic separations were carried out in a 2% agarose gel stained with SYBR Gold nucleic acid stain. Molecular weight markers: Lambda DNA digested with PstI (Lanes 1 and 4). (TIF) [file pone.0116822.s001.tif]

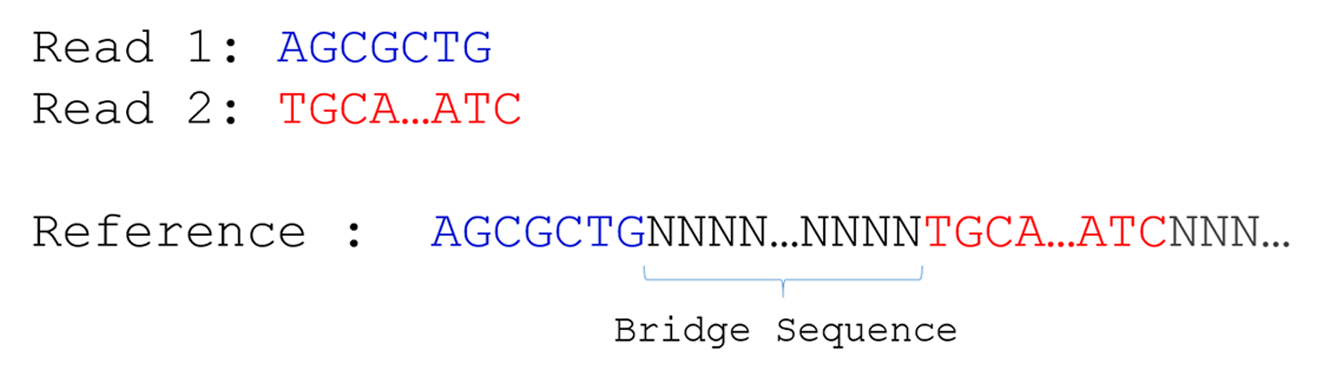

Supplement: S2 Fig — (TIF) [file pone.0116822.s002.tif]

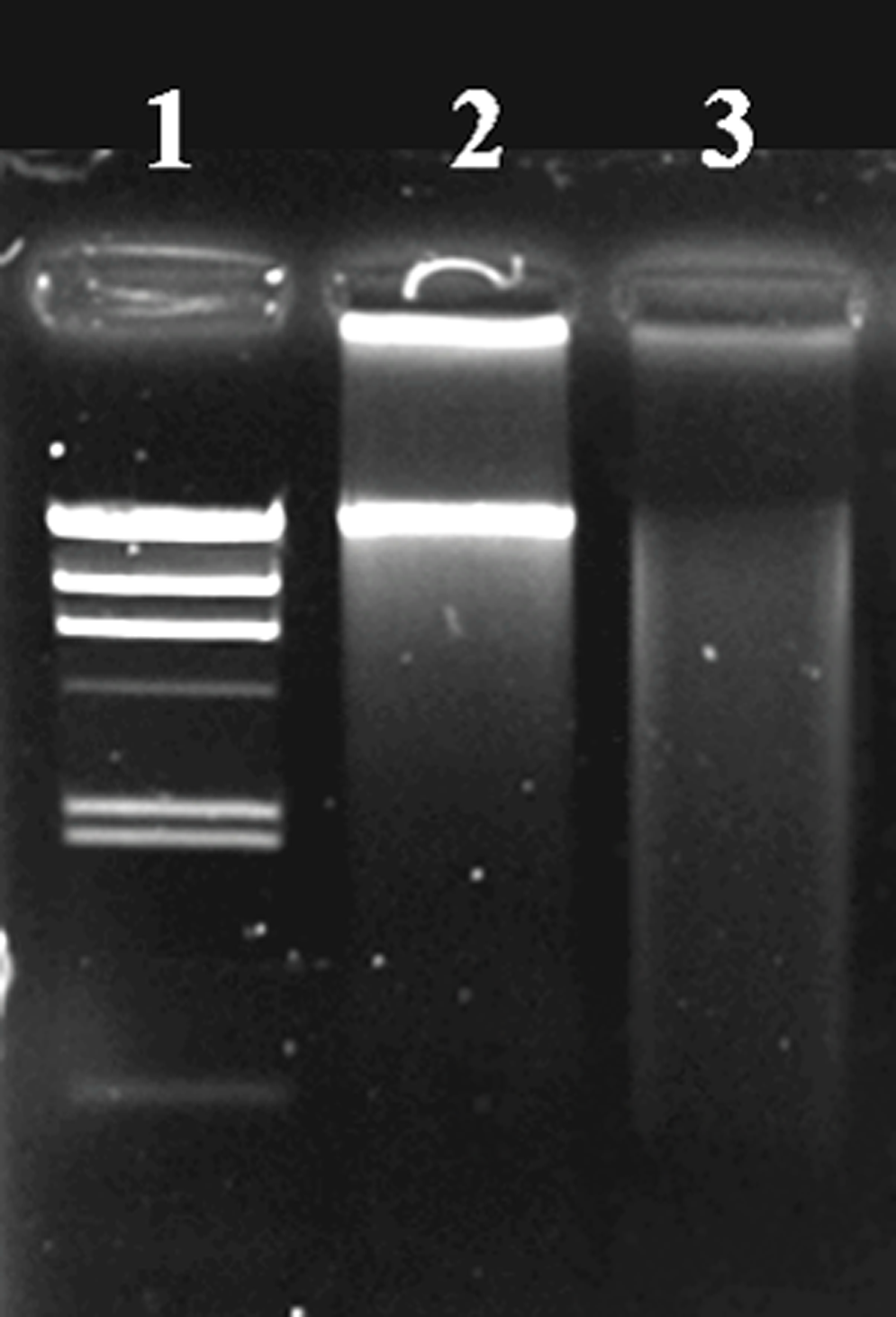

Supplement: S3 Fig — For size reference Lambda DNA digested with HindIII is shown in lane 1. (TIF) [file pone.0116822.s003.tif]

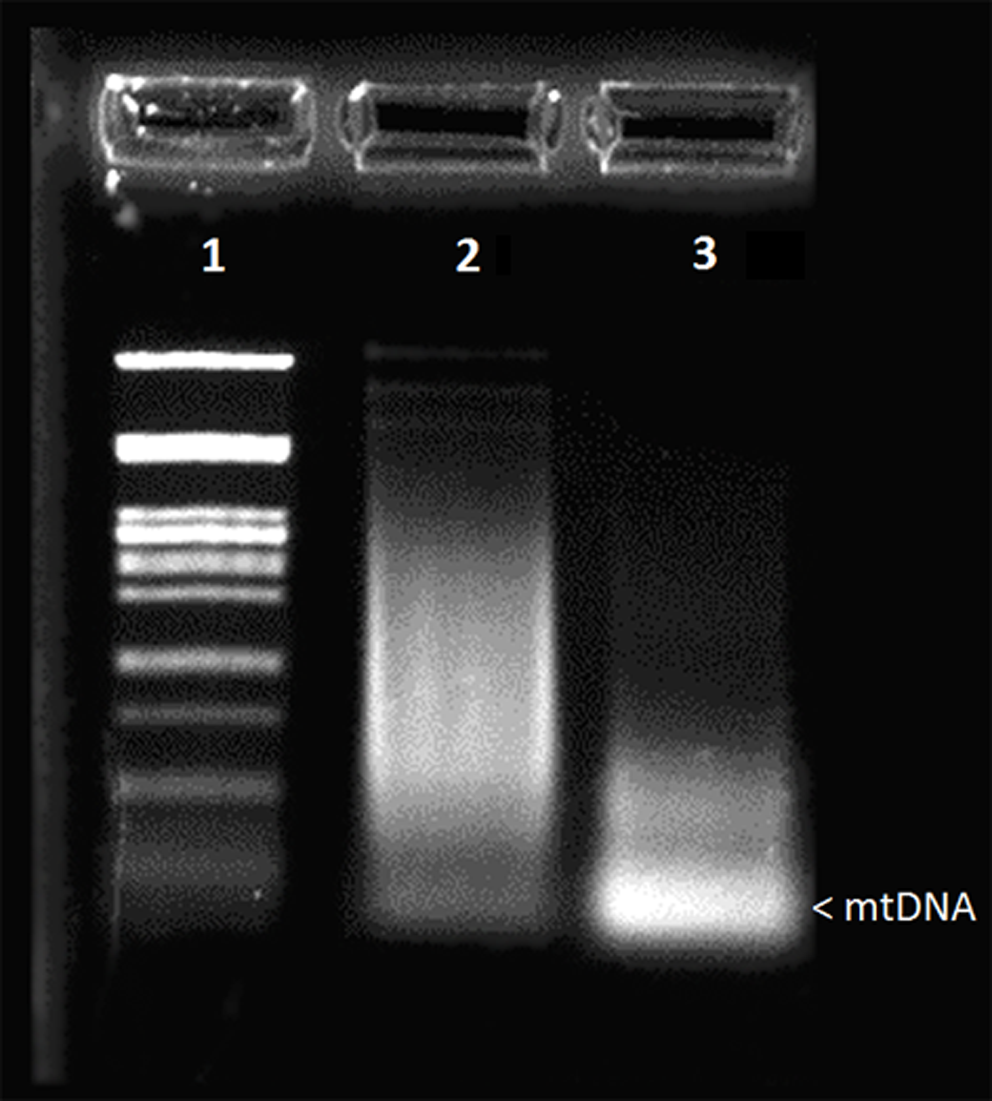

Supplement: S4 Fig — (1) PstI cut Lambda DNA (100ng), (2) PstI GBS library of Jamapa using purified nuclear DNA (3) Jamapa GBS library total DNA extractions (nDNA + mtDNA). (TIF) [file pone.0116822.s004.tif]

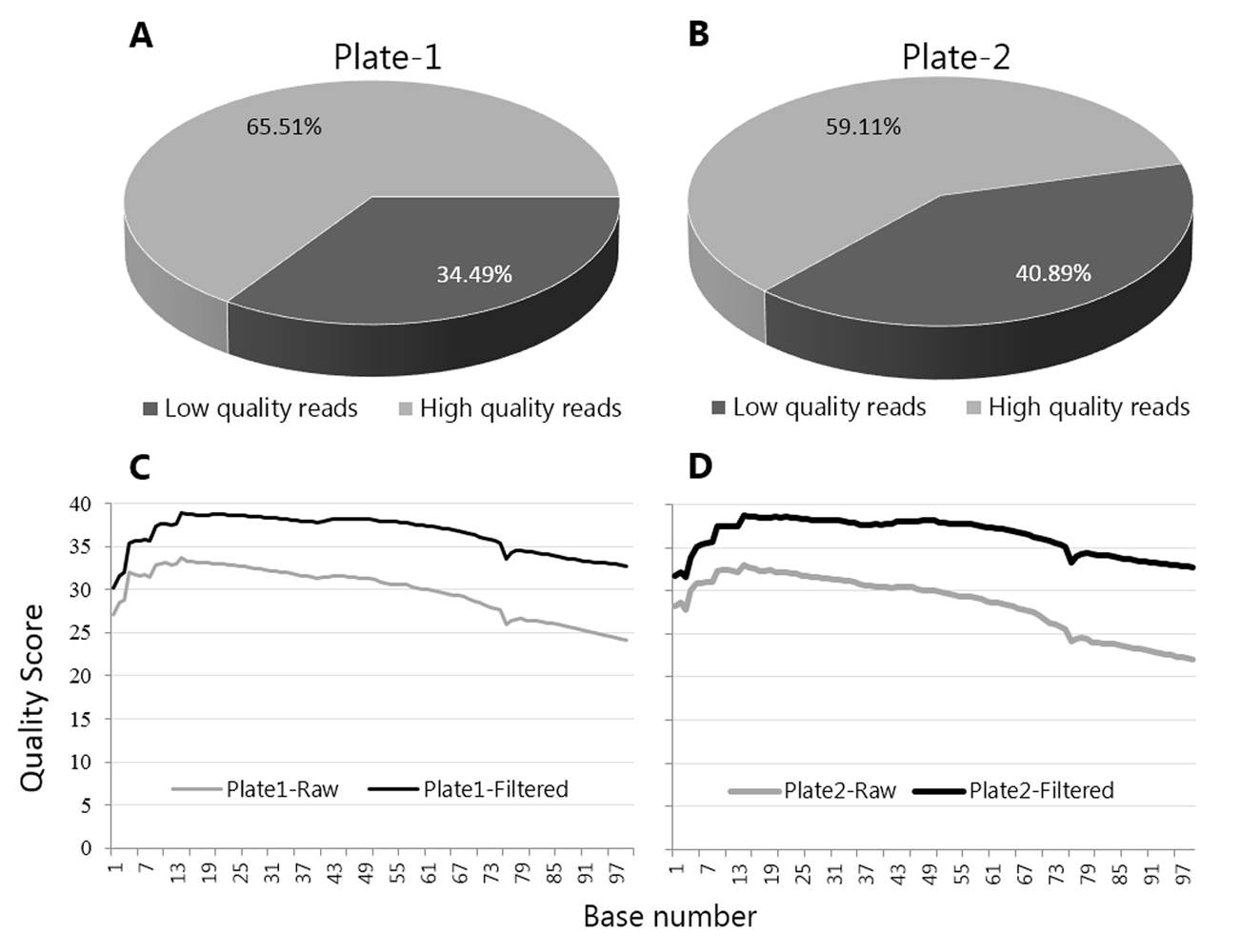

Supplement: S5 Fig — Improvement in quality score per base in each library after applying quality control filtration step (C, D). (TIF) [file pone.0116822.s005.tif]

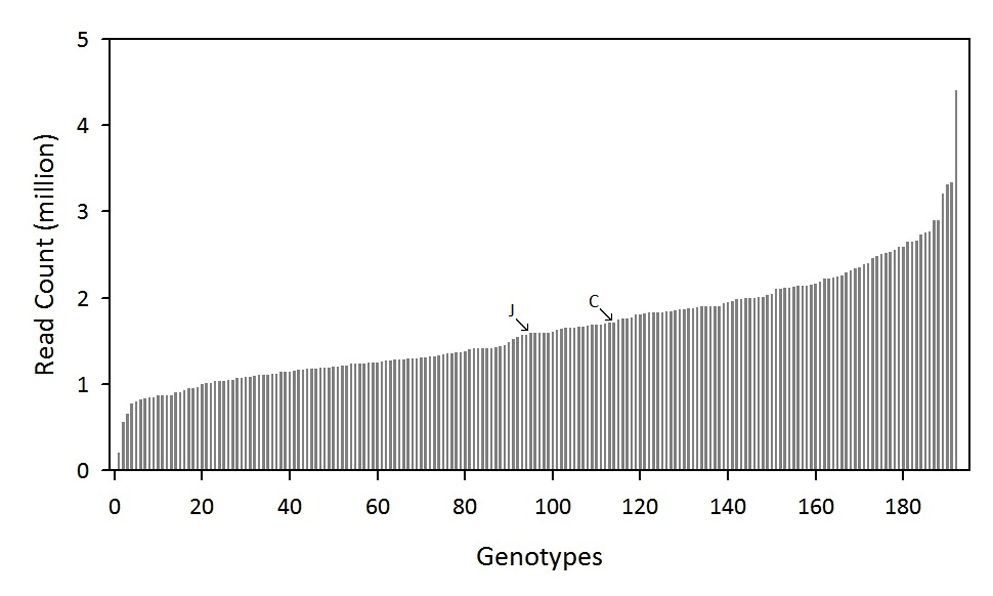

Supplement: S6 Fig — Average parental genotype reads count are represented by J (Jamapa) and C (Calima). (TIF) [file pone.0116822.s006.tif]

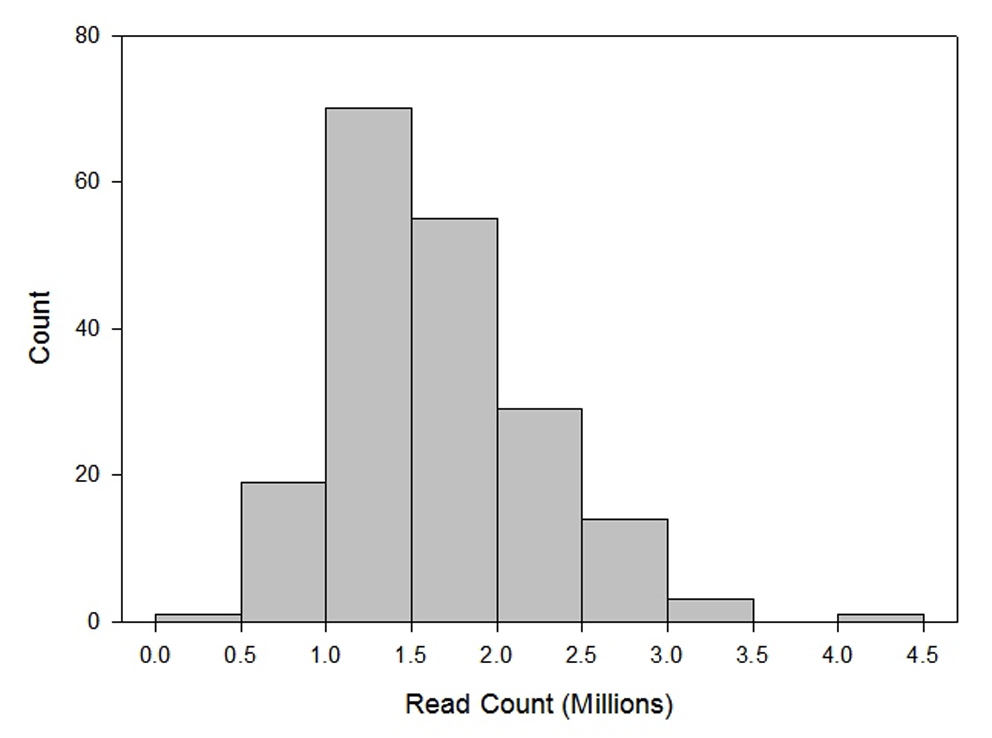

Supplement: S7 Fig — (TIF) [file pone.0116822.s007.tif]

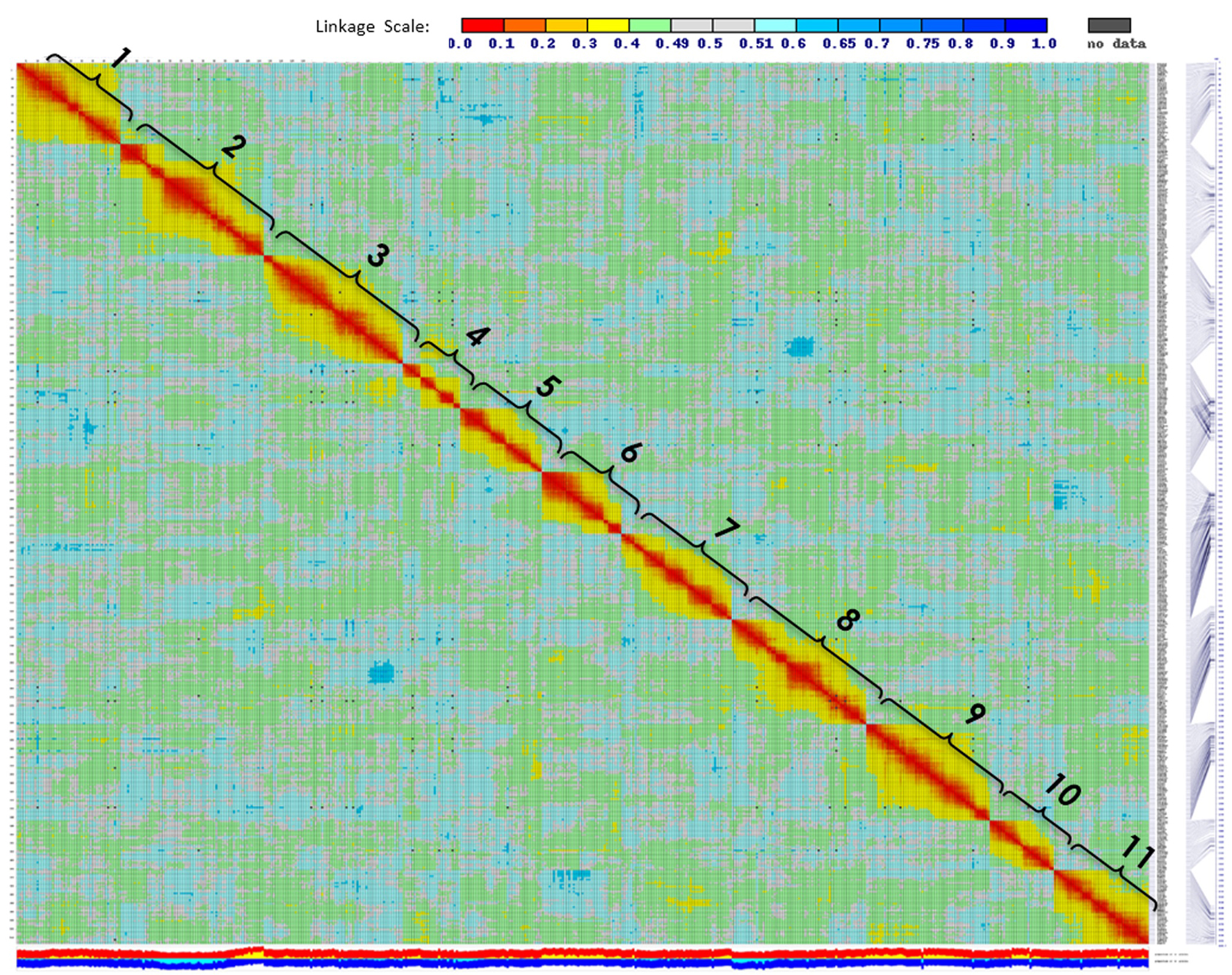

Supplement: S9 Fig — Strong linkage (red color) was observed between neighboring markers on the same chromosome; while weak/no linkage (green/blue) was observed between markers located on separate chromosomes. Yellow islands chromosomes. (TIF) [file pone.0116822.s009.tif]
