# Supplementary material for: Punctuated Distribution of Recombination Hotspots and Demarcation of Pericentromeric Regions in Phaseolus vulgaris L
Source: PLoS One. 2015 Jan 28;10(1):e0116822. doi: 10.1371/journal.pone.0116822 (PMC4309454; doi:10.1371/journal.pone.0116822)
Supplement: S1 Table — (DOCX) [file pone.0116822.s011.docx]

**Table S1. Brief specifications of mapped markers.**

| Chromosome | Marker | cM | Copy Number | TYPE |
| --- | --- | --- | --- | --- |
| Chr01 | DiM_1-1 | 0 | 1 | SNP |
| Chr01 | DiM_1-2 | 1.6 | 1 | SNP |
| Chr01 | Bng195 | 4.9 | 1 | RFLP |
| Chr01 | DiM_1-3 | 7.8 | 1 | SNP |
| Chr01 | DiM_1-4 | 11.4 | 1 | SNP |
| Chr01 | DiM_1-5 | 13.2 | 1 | SNP |
| Chr01 | DiM_1-6 | 14.2 | 1 | SNP |
| Chr01 | DiM_1-7 | 15.4 | 1 | SNP |
| Chr01 | DiM_1-8 | 17.1 | 1 | SNP |
| Chr01 | Bng072 | 17.8 | 1 | RFLP |
| Chr01 | DiM_1-9 | 19.5 | 1 | SNP |
| Chr01 | DiM_1-10 | 20.1 | 1 | SNP |
| Chr01 | DiM_1-11 | 20.7 | 4 | SNP |
| Chr01 | DiM_1-12 | 21.4 | 1 | SNP |
| Chr01 | DiM_1-13 | 22.1 | 1 | SNP |
| Chr01 | DiM_1-14 | 22.9 | 1 | SNP |
| Chr01 | DiM_1-15 | 26 | 1 | SNP |
| Chr01 | DiM_1-16 | 26.7 | 1 | SNP |
| Chr01 | DiM_1-17 | 28.1 | 1 | SNP |
| Chr01 | DiM_1-18 | 28.8 | 1 | SNP |
| Chr01 | DiM_1-19 | 30.6 | 1 | SNP |
| Chr01 | DiM_1-20 | 31.9 | 1 | SNP |
| Chr01 | DiM_1-21 | 33.4 | 1 | SNP |
| Chr01 | DiM_1-22 | 37.2 | 1 | SNP |
| Chr01 | DiM_1-23 | 40 | 1 | SNP |
| Chr01 | DiM_1-24 | 40.3 | 1 | SNP |
| Chr01 | DiM_1-25 | 40.6 | 1 | SNP |
| Chr01 | Fin | 42.1 | NA | Phenotype |
| Chr01 | DiM_1-26 | 49.4 | 1 | SNP |
| Chr01 | DiM_1-27 | 50.1 | 1 | SNP |
| Chr01 | Bng227 | 53.5 | 1 | RFLP |
| Chr01 | DiM_1-28 | 58.8 | 1 | SNP |
| Chr01 | DiM_1-29 | 60 | 1 | SNP |
| Chr01 | DiM_1-30 | 62.4 | 1 | SNP |
| Chr01 | DiM_1-31 | 63.6 | 1 | SNP |
| Chr01 | Bng126 | 65.8 | 1 | RFLP |
| Chr01 | DiM_1-32 | 67.9 | 1 | SNP |
| Chr01 | DiM_1-33 | 68.5 | 1 | SNP |
| Chr01 | DiM_1-34 | 70 | 1 | SNP |
| Chr01 | DiM_1-35 | 71.6 | 1 | SNP |
| Chr01 | DiM_1-36 | 72.8 | 1 | SNP |
| Chr01 | Mips1 | 74.1 | 1 | RFLP |
| Chr01 | DiM_1-37 | 75.2 | 1 | SNP |
| Chr01 | DiM_1-38 | 76.5 | 1 | SNP |
| Chr01 | Hg1.1SDfr | 78.2 | 1 | SNP |
| Chr01 | DiM_1-39 | 80.8 | 1 | SNP |
| Chr01 | Bng083 | 87.1 | 1 | RFLP |
| Chr02 | Bng057 | 0 | 1 | RFLP |
| Chr02 | DiM_2-1 | 2.1 | 1 | SNP |
| Chr02 | DiM_2-2 | 3 | 1 | SNP |
| Chr02 | DiM_2-3 | 4.4 | 1 | SNP |
| Chr02 | DiM_2-4 | 4.8 | 1 | SNP |
| Chr02 | DiM_2-5 | 5.3 | 1 | SNP |
| Chr02 | DiM_2-6 | 6 | 1 | SNP |
| Chr02 | DiM_2-7 | 6.8 | 1 | SNP |
| Chr02 | DiM_2-8 | 8.1 | 1 | SNP |
| Chr02 | Bng061 | 11.5 | 1 | RFLP |
| Chr02 | Bng074 | 15.1 | 1 | RFLP |
| Chr02 | DiM_2-9 | 20.4 | 1 | SNP |
| Chr02 | DiM_2-10 | 27.7 | 1 | SNP |
| Chr02 | DiM_2-11 | 29.8 | 1 | SNP |
| Chr02 | DiM_2-12 | 35.7 | 1 | SNP |
| Chr02 | DiM_2-13 | 36.8 | 1 | SNP |
| Chr02 | DiM_2-14 | 38.9 | 1 | SNP |
| Chr02 | DiM_2-15 | 39.5 | 1 | SNP |
| Chr02 | Bng098 | 42.3 | 1 | RFLP |
| Chr02 | DiM_2-16 | 44.7 | 1 | SNP |
| Chr02 | DiM_2-17 | 49 | 1 | SNP |
| Chr02 | DiM_2-18 | 49.9 | 1 | SNP |
| Chr02 | DiM_2-19 | 50.8 | 1 | SNP |
| Chr02 | DiM_2-20 | 51.6 | 1 | SNP |
| Chr02 | DiM_2-21 | 52.4 | 1 | SNP |
| Chr02 | DiM_2-22 | 52.7 | 1 | SNP |
| Chr02 | DiM_2-23 | 53.3 | 1 | SNP |
| Chr02 | DiM_2-24 | 53.9 | 1 | SNP |
| Chr02 | DiM_2-25 | 54.2 | 1 | SNP |
| Chr02 | DiM_2-26 | 55.2 | 1 | SNP |
| Chr02 | DiM_2-27 | 55.9 | 1 | SNP |
| Chr02 | DiM_2-28 | 56.6 | 1 | SNP |
| Chr02 | DiM_2-29 | 58.6 | 1 | SNP |
| Chr02 | DiM_2-30 | 60.3 | 1 | SNP |
| Chr02 | DiM_2-31 | 61.8 | 1 | SNP |
| Chr02 | DiM_2-32 | 62.3 | 1 | SNP |
| Chr02 | DiM_2-33 | 63.4 | 1 | SNP |
| Chr02 | DiM_2-34 | 64.8 | 1 | SNP |
| Chr02 | DiM_2-35 | 66.2 | 1 | SNP |
| Chr02 | DiM_2-36 | 68.1 | 1 | SNP |
| Chr02 | Bng082 | 69.7 | 1 | RFLP |
| Chr02 | DiM_2-37 | 74.1 | 1 | SNP |
| Chr02 | DiM_2-38 | 75 | 1 | SNP |
| Chr02 | DiM_2-39 | 75.7 | 1 | SNP |
| Chr02 | DiM_2-40 | 81.2 | 1 | SNP |
| Chr02 | DiM_2-41 | 82.1 | 1 | SNP |
| Chr02 | DiM_2-42 | 82.7 | 1 | SNP |
| Chr02 | Bng108 | 84.5 | 1 | RFLP |
| Chr02 | DiM_2-43 | 89.2 | 1 | SNP |
| Chr02 | DiM_2-44 | 89.7 | 1 | SNP |
| Chr02 | DiM_2-45 | 91.7 | 1 | SNP |
| Chr02 | DiM_2-46 | 95.6 | 1 | SNP |
| Chr02 | DiM_2-47 | 98.1 | 1 | SNP |
| Chr02 | DiM_2-48 | 99.4 | 1 | SNP |
| Chr02 | DiM_2-49 | 101.4 | 1 | SNP |
| Chr02 | Bng141 | 105.1 | 1 | RFLP |
| Chr02 | DiM_2-50 | 107.4 | 1 | SNP |
| Chr02 | DiM_2-51 | 109.4 | 1 | SNP |
| Chr02 | DiM_2-52 | 110.2 | 1 | SNP |
| Chr02 | DiM_2-53 | 110.6 | 1 | SNP |
| Chr02 | DiM_2-54 | 111.7 | 1 | SNP |
| Chr02 | DiM_2-55 | 112.6 | 1 | SNP |
| Chr02 | DiM_2-56 | 113.8 | 1 | SNP |
| Chr02 | DiM_2-57 | 115.8 | 1 | SNP |
| Chr02 | DGrpU | 119.9 | 1 | SNP |
| Chr03 | DiM_3-1 | 0 | 1 | SNP |
| Chr03 | DiM_3-2 | 1.1 | 1 | SNP |
| Chr03 | DiM_3-3 | 2.3 | 1 | SNP |
| Chr03 | DiM_3-4 | 4.5 | 1 | SNP |
| Chr03 | DiM_3-5 | 11.7 | 1 | SNP |
| Chr03 | DiM_3-6 | 14 | 1 | SNP |
| Chr03 | DiM_3-7 | 16.1 | 1 | SNP |
| Chr03 | DiM_3-8 | 17.6 | 1 | SNP |
| Chr03 | DiM_3-9 | 19.7 | 1 | SNP |
| Chr03 | Bng012 | 22 | 1 | RFLP |
| Chr03 | DiM_3-10 | 23 | 1 | SNP |
| Chr03 | DiM_3-11 | 24 | 1 | SNP |
| Chr03 | DiM_3-12 | 26.8 | 1 | SNP |
| Chr03 | DiM_3-13 | 27.6 | 1 | SNP |
| Chr03 | DiM_3-14 | 28.4 | 1 | SNP |
| Chr03 | DiM_3-15 | 29.15 | 1 | SNP |
| Chr03 | DiM_3-16 | 29.9 | 1 | SNP |
| Chr03 | DiM_3-17 | 31.3 | 1 | SNP |
| Chr03 | DiM_3-18 | 32.1 | 1 | SNP |
| Chr03 | DiM_3-19 | 33.4 | 1 | SNP |
| Chr03 | DiM_3-20 | 34.3 | 1 | SNP |
| Chr03 | Bng114 | 35.5 | 1 | RFLP |
| Chr03 | DiM_3-21 | 37.2 | 1 | SNP |
| Chr03 | DiM_3-22 | 38.2 | 1 | SNP |
| Chr03 | DiM_3-23 | 39 | 1 | SNP |
| Chr03 | DiM_3-24 | 39.7 | 1 | SNP |
| Chr03 | Bng123 | 40.6 | 1 | RFLP |
| Chr03 | DiM_3-25 | 41.8 | 0 | SNP |
| Chr03 | DiM_3-26 | 44.2 | 1 | SNP |
| Chr03 | Bng164 | 47.4 | 1 | RFLP |
| Chr03 | DiM_3-27 | 49.2 | 1 | SNP |
| Chr03 | DiM_3-28 | 53.1 | 1 | SNP |
| Chr03 | DiM_3-29 | 56.3 | 1 | SNP |
| Chr03 | DiM_3-30 | 58.1 | 1 | SNP |
| Chr03 | DiM_3-31 | 59.3 | 1 | SNP |
| Chr03 | DiM_3-32 | 60.4 | 1 | SNP |
| Chr03 | DiM_3-33 | 61.4 | 1 | SNP |
| Chr03 | Bng242 | 62 | 1 | RFLP |
| Chr03 | DiM_3-34 | 62.6 | 1 | SNP |
| Chr03 | Bng165 | 63.1 | 1 | RFLP |
| Chr03 | Bng155 | 64.1 | 1 | RFLP |
| Chr03 | DiM_3-35 | 65.6 | 2 | SNP |
| Chr03 | DiM_3-36 | 66.4 | 1 | SNP |
| Chr03 | DiM_3-37 | 66.8 | 1 | SNP |
| Chr03 | DiM_3-38 | 67.2 | 1 | SNP |
| Chr03 | DiM_3-39 | 68.7 | 1 | SNP |
| Chr03 | DiM_3-40 | 71.8 | 1 | SNP |
| Chr03 | Bng216 | 74.2 | 1 | RFLP |
| Chr03 | DiM_3-41 | 76 | 1 | SNP |
| Chr03 | DiM_3-42 | 76.8 | 1 | SNP |
| Chr03 | DiM_3-43 | 78.4 | 1 | SNP |
| Chr03 | DiM_3-44 | 79.3 | 1 | SNP |
| Chr03 | DiM_3-45 | 82.2 | 1 | SNP |
| Chr03 | DiM_3-46 | 82.9 | 1 | SNP |
| Chr03 | DiM_3-47 | 85.9 | 1 | SNP |
| Chr03 | Bng033 | 86.9 | 1 | RFLP |
| Chr03 | DiM_3-48 | 89.9 | 1 | SNP |
| Chr03 | DiM_3-49 | 90.9 | 1 | SNP |
| Chr03 | DiM_3-50 | 91.8 | 1 | SNP |
| Chr03 | DiM_3-51 | 93.5 | 1 | SNP |
| Chr03 | DiM_3-52 | 96.8 | 1 | SNP |
| Chr03 | Bng021 | 102 | 1 | RFLP |
| Chr03 | Bng250 | 103.1 | 1 | RFLP |
| Chr04 | DiM_4-1 | 0 | 1 | SNP |
| Chr04 | DiM_4-2 | 1.9 | 1 | SNP |
| Chr04 | DiM_4-3 | 4 | 1 | SNP |
| Chr04 | DiM_4-4 | 13 | 1 | SNP |
| Chr04 | DiM_4-5 | 13.9 | 1 | SNP |
| Chr04 | DiM_4-6 | 15 | 1 | SNP |
| Chr04 | DiM_4-7 | 16 | 1 | SNP |
| Chr04 | DiM_4-8 | 17 | 1 | SNP |
| Chr04 | DiM_4-9 | 18.2 | 1 | SNP |
| Chr04 | DiM_4-10 | 22.9 | 1 | SNP |
| Chr04 | DiM_4-11 | 30.6 | 1 | SNP |
| Chr04 | DiM_4-12 | 38 | 1 | SNP |
| Chr04 | DiM_4-13 | 42.2 | 1 | SNP |
| Chr04 | DiM_4-14 | 42.9 | 1 | SNP |
| Chr04 | DiM_4-15 | 44.6 | 1 | SNP |
| Chr04 | DiM_4-16 | 46.2 | 1 | SNP |
| Chr04 | DiM_4-17 | 49 | 1 | SNP |
| Chr04 | DiM_4-18 | 50.1 | 1 | SNP |
| Chr04 | DiM_4-19 | 63.4 | 0 | SNP |
| Chr04 | DiM_4-20 | 64.3 | 1 | SNP |
| Chr04 | Bng224 | 67.1 | 1 | RFLP |
| Chr04 | DiM_4-21 | 69.4 | 1 | SNP |
| Chr04 | DiM_4-22 | 70.4 | 1 | SNP |
| Chr04 | DiM_4-23 | 72.1 | 1 | SNP |
| Chr04 | DiM_4-24 | 74.5 | 1 | SNP |
| Chr04 | DiM_4-25 | 78 | 1 | SNP |
| Chr05 | DiM_5-1 | 0 | 1 | SNP |
| Chr05 | DiM_5-2 | 2.6 | 1 | SNP |
| Chr05 | DiM_5-3 | 6.9 | 1 | SNP |
| Chr05 | DiM_5-4 | 9.7 | 1 | SNP |
| Chr05 | DiM_5-5 | 12.7 | 1 | SNP |
| Chr05 | DiM_5-6 | 19.1 | 1 | SNP |
| Chr05 | DiM_5-7 | 20.6 | 1 | SNP |
| Chr05 | DiM_5-8 | 21.9 | 1 | SNP |
| Chr05 | DiM_5-9 | 22.8 | 1 | SNP |
| Chr05 | DiM_5-10 | 24.3 | 1 | SNP |
| Chr05 | DiM_5-11 | 25.3 | 1 | SNP |
| Chr05 | DiM_5-12 | 27.4 | 1 | SNP |
| Chr05 | DiM_5-13 | 28.6 | 1 | SNP |
| Chr05 | DiM_5-14 | 30.9 | 1 | SNP |
| Chr05 | DiM_5-15 | 32.4 | 1 | SNP |
| Chr05 | Bng205c | 33.9 | 1 | RFLP |
| Chr05 | DiM_5-16 | 37.2 | 1 | SNP |
| Chr05 | DiM_5-17 | 41.1 | 1 | SNP |
| Chr05 | DiM_5-18 | 42 | 1 | SNP |
| Chr05 | DiM_5-19 | 43.4 | 1 | SNP |
| Chr05 | DiM_5-20 | 44.3 | 1 | SNP |
| Chr05 | DiM_5-21 | 46.6 | 1 | SNP |
| Chr05 | Bng133 | 47.6 | 1 | RFLP |
| Chr05 | DiM_5-22 | 52.7 | 1 | SNP |
| Chr05 | DiM_5-23 | 55.1 | 1 | SNP |
| Chr05 | DiM_5-24 | 57.1 | 1 | SNP |
| Chr05 | DiM_5-25 | 59.2 | 1 | SNP |
| Chr05 | DiM_5-26 | 61.7 | 1 | SNP |
| Chr05 | DiM_5-27 | 62.6 | 1 | SNP |
| Chr05 | DiM_5-28 | 62.9 | 1 | SNP |
| Chr05 | DiM_5-29 | 63.4 | 1 | SNP |
| Chr05 | DiM_5-30 | 64.2 | 1 | SNP |
| Chr05 | DiM_5-31 | 64.5 | 2 | SNP |
| Chr05 | DiM_5-32 | 64.8 | 1 | SNP |
| Chr05 | DiM_5-33 | 66.2 | 1 | SNP |
| Chr05 | DiM_5-34 | 69.3 | 1 | SNP |
| Chr05 | Bng162 | 71.8 | 1 | RFLP |
| Chr06 | Bng026 | 0 | 1 | RFLP |
| Chr06 | DiM_6-1 | 2 | 1 | SNP |
| Chr06 | DiM_6-2 | 3.4 | 1 | SNP |
| Chr06 | DiM_6-3 | 3.9 | 1 | SNP |
| Chr06 | DiM_6-4 | 4.4 | 1 | SNP |
| Chr06 | DiM_6-5 | 5.4 | 1 | SNP |
| Chr06 | DiM_6-6 | 5.9 | 1 | SNP |
| Chr06 | DiM_6-7 | 6.2 | 1 | SNP |
| Chr06 | DiM_6-8 | 6.9 | 1 | SNP |
| Chr06 | DiM_6-9 | 7.6 | 1 | SNP |
| Chr06 | DiM_6-10 | 8.9 | 1 | SNP |
| Chr06 | DiM_6-11 | 12 | 1 | SNP |
| Chr06 | DiM_6-12 | 12.5 | 1 | SNP |
| Chr06 | DiM_6-13 | 13.6 | 1 | SNP |
| Chr06 | DiM_6-14 | 16 | 1 | SNP |
| Chr06 | DiM_6-15 | 17.4 | 1 | SNP |
| Chr06 | Bng088 | 18.7 | 1 | RFLP |
| Chr06 | DiM_6-16 | 21.7 | 1 | SNP |
| Chr06 | DiM_6-17 | 23.2 | 1 | SNP |
| Chr06 | Bng009 | 25.4 | 1 | RFLP |
| Chr06 | DiM_6-18 | 26 | 1 | SNP |
| Chr06 | DiM_6-19 | 27 | 1 | SNP |
| Chr06 | DiM_6-20 | 27.7 | 1 | SNP |
| Chr06 | Bng027 | 28.8 | 1 | RFLP |
| Chr06 | DiM_6-21 | 30.7 | 1 | SNP |
| Chr06 | DiM_6-22 | 31.3 | 1 | SNP |
| Chr06 | DiM_6-23 | 32 | 1 | SNP |
| Chr06 | Bng252 | 33.9 | 1 | RFLP |
| Chr06 | DiM_6-24 | 42.7 | 1 | SNP |
| Chr06 | Bng183 | 44.2 | 1 | RFLP |
| Chr06 | DiM_6-25 | 52.4 | 1 | SNP |
| Chr06 | DiM_6-26 | 54.5 | 1 | SNP |
| Chr06 | DiM_6-27 | 55.5 | 1 | SNP |
| Chr06 | DiM_6-28 | 55.8 | 1 | SNP |
| Chr06 | Bng087 | 56.1 | 1 | RFLP |
| Chr06 | DiM_6-29 | 59.2 | 1 | SNP |
| Chr07 | Bng168 | 0 | 1 | RFLP |
| Chr07 | DiM_7-1 | 2.2 | 1 | SNP |
| Chr07 | DiM_7-2 | 6.7 | 1 | SNP |
| Chr07 | DiM_7-3 | 7.6 | 1 | SNP |
| Chr07 | Bng249 | 11.7 | 1 | RFLP |
| Chr07 | DiM_7-4 | 14.6 | 1 | SNP |
| Chr07 | DiM_7-5 | 21.2 | 1 | SNP |
| Chr07 | DiM_7-6 | 23.2 | 1 | SNP |
| Chr07 | CycD | 26.2 | 1 | SNP |
| Chr07 | Bng042 | 28.9 | 1 | RFLP |
| Chr07 | DiM_7-7 | 34.4 | 1 | SNP |
| Chr07 | DiM_7-8 | 37.2 | 1 | SNP |
| Chr07 | DiM_7-9 | 39.9 | 2 | SNP |
| Chr07 | DiM_7-10 | 42.8 | 1 | SNP |
| Chr07 | DiM_7-11 | 44.7 | 1 | SNP |
| Chr07 | DiM_7-12 | 45.9 | 1 | SNP |
| Chr07 | DiM_7-13 | 47.7 | 1 | SNP |
| Chr07 | DiM_7-14 | 49.2 | 1 | SNP |
| Chr07 | DiM_7-15 | 50 | 1 | SNP |
| Chr07 | DiM_7-16 | 52.7 | 1 | SNP |
| Chr07 | Bng244 | 57.1 | 1 | RFLP |
| Chr07 | DiM_7-17 | 59.5 | 1 | SNP |
| Chr07 | DiM_7-18 | 60.2 | 1 | SNP |
| Chr07 | DiM_7-19 | 60.6 | 1 | SNP |
| Chr07 | DiM_7-20 | 61.6 | 1 | SNP |
| Chr07 | DiM_7-21 | 62.3 | 1 | SNP |
| Chr07 | DiM_7-22 | 63.3 | 1 | SNP |
| Chr07 | DiM_7-23 | 64.3 | 1 | SNP |
| Chr07 | DiM_7-24 | 67.4 | 1 | SNP |
| Chr07 | Bng203 | 70.5 | 1 | RFLP |
| Chr07 | DiM_7-25 | 75.3 | 1 | SNP |
| Chr07 | DiM_7-26 | 77.3 | 1 | SNP |
| Chr07 | DiM_7-27 | 78.9 | 1 | SNP |
| Chr07 | DiM_7-28 | 81.1 | 1 | SNP |
| Chr07 | Bng118 | 82.5 | 1 | RFLP |
| Chr07 | DiM_7-29 | 83.3 | 1 | SNP |
| Chr07 | DiM_7-30 | 84.2 | 1 | SNP |
| Chr07 | DiM_7-31 | 85.3 | 1 | SNP |
| Chr07 | DiM_7-32 | 85.7 | 1 | SNP |
| Chr07 | DiM_7-33 | 86.4 | 1 | SNP |
| Chr07 | DiM_7-34 | 87.5 | 1 | SNP |
| Chr07 | DiM_7-35 | 92.2 | 1 | SNP |
| Chr07 | DiM_7-36 | 93.8 | 1 | SNP |
| Chr07 | DiM_7-37 | 95.5 | 1 | SNP |
| Chr07 | DiM_7-38 | 97.4 | 1 | SNP |
| Chr07 | DiM_7-39 | 98.7 | 1 | SNP |
| Chr07 | Bng047 | 99.9 | 1 | RFLP |
| Chr07 | DiM_7-40 | 101.4 | 1 | SNP |
| Chr07 | DiM_7-41 | 106.8 | 1 | SNP |
| Chr07 | DiM_7-42 | 108.9 | 1 | SNP |
| Chr08 | Bng139 | 0 | 1 | RFLP |
| Chr08 | DiM_8-1 | 5.2 | 1 | SNP |
| Chr08 | DiM_8-2 | 7.2 | 1 | SNP |
| Chr08 | DiM_8-3 | 12.5 | 1 | SNP |
| Chr08 | DiM_8-4 | 13.2 | 1 | SNP |
| Chr08 | DiM_8-5 | 13.5 | 1 | SNP |
| Chr08 | DiM_8-6 | 13.8 | 1 | SNP |
| Chr08 | DiM_8-7 | 15.6 | 1 | SNP |
| Chr08 | DiM_8-8 | 16.7 | 1 | SNP |
| Chr08 | Bng069 | 17.8 | 1 | RFLP |
| Chr08 | DiM_8-9 | 19.9 | 1 | SNP |
| Chr08 | Bng128 | 21.8 | 1 | RFLP |
| Chr08 | StdPig | 26 | NA | Phenotype |
| Chr08 | DiM_8-10 | 30 | 1 | SNP |
| Chr08 | DiM_8-11 | 31.2 | 1 | SNP |
| Chr08 | DiM_8-12 | 32.7 | 1 | SNP |
| Chr08 | Bng062 | 34.9 | 1 | RFLP |
| Chr08 | DiM_8-13 | 37.5 | 1 | SNP |
| Chr08 | DiM_8-14 | 39.1 | 1 | SNP |
| Chr08 | DiM_8-15 | 41.7 | 1 | SNP |
| Chr08 | DiM_8-16 | 44.7 | 1 | SNP |
| Chr08 | DiM_8-17 | 45.4 | 1 | SNP |
| Chr08 | Bng007 | 46.9 | 1 | RFLP |
| Chr08 | DiM_8-18 | 49.4 | 1 | SNP |
| Chr08 | DiM_8-19 | 51.7 | 1 | SNP |
| Chr08 | DiM_8-20 | 53.4 | 1 | SNP |
| Chr08 | DiM_8-21 | 54.9 | 1 | SNP |
| Chr08 | DiM_8-22 | 55.6 | 1 | SNP |
| Chr08 | DiM_8-23 | 55.9 | 1 | SNP |
| Chr08 | DiM_8-24 | 56.9 | 1 | SNP |
| Chr08 | DiM_8-25 | 58 | 1 | SNP |
| Chr08 | DiM_8-26 | 58.6 | 1 | SNP |
| Chr08 | DiM_8-27 | 59.7 | 1 | SNP |
| Chr08 | DiM_8-28 | 60.1 | 1 | SNP |
| Chr08 | DiM_8-29 | 61.6 | 1 | SNP |
| Chr08 | DiM_8-30 | 63.2 | 1 | SNP |
| Chr08 | DiM_8-31 | 67.5 | 1 | SNP |
| Chr08 | DiM_8-32 | 69.5 | 1 | SNP |
| Chr08 | DiM_8-33 | 69.9 | 1 | SNP |
| Chr08 | DiM_8-34 | 70.8 | 1 | SNP |
| Chr08 | DiM_8-35 | 71.8 | 1 | SNP |
| Chr08 | DiM_8-36 | 77 | 1 | SNP |
| Chr08 | DiM_8-37 | 77.7 | 1 | SNP |
| Chr08 | DiM_8-38 | 81 | 1 | SNP |
| Chr08 | DiM_8-39 | 82 | 1 | SNP |
| Chr08 | DiM_8-40 | 87.6 | 1 | SNP |
| Chr08 | DiM_8-41 | 89.5 | 1 | SNP |
| Chr08 | DiM_8-42 | 91.1 | 1 | SNP |
| Chr08 | DiM_8-43 | 91.9 | 1 | SNP |
| Chr08 | GG3.2SD1 | 93.3 | 1 | SNP |
| Chr08 | DiM_8-44 | 94 | 1 | SNP |
| Chr08 | DiM_8-45 | 95.7 | 1 | SNP |
| Chr08 | DiM_8-46 | 96.8 | 1 | SNP |
| Chr08 | DiM_8-47 | 97.6 | 1 | SNP |
| Chr08 | DiM_8-48 | 100 | 1 | SNP |
| Chr08 | Bng131 | 100.8 | 1 | RFLP |
| Chr08 | DiM_8-49 | 102.3 | 1 | SNP |
| Chr08 | DiM_8-50 | 103.9 | 1 | SNP |
| Chr08 | DiM_8-51 | 105.7 | 1 | SNP |
| Chr08 | DiM_8-52 | 108.7 | 1 | SNP |
| Chr08 | DiM_8-53 | 111.4 | 1 | SNP |
| Chr09 | DiM_9-1 | 0 | 1 | SNP |
| Chr09 | DiM_9-2 | 0.6 | 1 | SNP |
| Chr09 | DiM_9-3 | 1.2 | 1 | SNP |
| Chr09 | DiM_9-4 | 1.5 | 1 | SNP |
| Chr09 | DiM_9-5 | 2.7 | 1 | SNP |
| Chr09 | DiM_9-6 | 5 | 1 | SNP |
| Chr09 | DiM_9-7 | 5.9 | 1 | SNP |
| Chr09 | DiM_9-8 | 8.1 | 1 | SNP |
| Chr09 | Bng035 | 10.4 | 1 | RFLP |
| Chr09 | DiM_9-9 | 11 | 1 | SNP |
| Chr09 | DiM_9-10 | 11.6 | 1 | SNP |
| Chr09 | Bng181 | 12.8 | 1 | RFLP |
| Chr09 | DiM_9-11 | 13.7 | 1 | SNP |
| Chr09 | DiM_9-12 | 15.8 | 1 | SNP |
| Chr09 | DiM_9-13 | 16.8 | 1 | SNP |
| Chr09 | Bng163 | 18.3 | 1 | RFLP |
| Chr09 | DiM_9-14 | 20.2 | 1 | SNP |
| Chr09 | DiM_9-15 | 21.9 | 1 | SNP |
| Chr09 | Bng176 | 24.7 | 1 | RFLP |
| Chr09 | DiM_9-16 | 25.3 | 1 | SNP |
| Chr09 | DiM_9-17 | 25.6 | 1 | SNP |
| Chr09 | DiM_9-18 | 25.9 | 1 | SNP |
| Chr09 | DiM_9-19 | 27.7 | 1 | SNP |
| Chr09 | DiM_9-20 | 28.4 | 1 | SNP |
| Chr09 | DiM_9-21 | 29.3 | 1 | SNP |
| Chr09 | DiM_9-22 | 30.1 | 1 | SNP |
| Chr09 | DiM_9-23 | 31.9 | 1 | SNP |
| Chr09 | DiM_9-24 | 32.5 | 1 | SNP |
| Chr09 | DiM_9-25 | 33.5 | 1 | SNP |
| Chr09 | DiM_9-26 | 35.8 | 1 | SNP |
| Chr09 | DiM_9-27 | 36.5 | 1 | SNP |
| Chr09 | DiM_9-28 | 37.8 | 1 | SNP |
| Chr09 | DiM_9-29 | 39.3 | 1 | SNP |
| Chr09 | DiM_9-30 | 41.1 | 1 | SNP |
| Chr09 | DiM_9-31 | 41.6 | 1 | SNP |
| Chr09 | DiM_9-32 | 43.8 | 1 | SNP |
| Chr09 | DiM_9-33 | 45.4 | 1 | SNP |
| Chr09 | DiM_9-34 | 46 | 1 | SNP |
| Chr09 | DiM_9-35 | 46.6 | 1 | SNP |
| Chr09 | DiM_9-36 | 48.1 | 1 | SNP |
| Chr09 | DiM_9-37 | 50.2 | 1 | SNP |
| Chr09 | DiM_9-38 | 51.7 | 1 | SNP |
| Chr09 | DiM_9-39 | 52.1 | 1 | SNP |
| Chr09 | DiM_9-40 | 53.6 | 1 | SNP |
| Chr09 | DiM_9-41 | 54.6 | 1 | SNP |
| Chr09 | DiM_9-42 | 57.1 | 1 | SNP |
| Chr09 | DiM_9-43 | 57.7 | 1 | SNP |
| Chr09 | DiM_9-44 | 61 | 1 | SNP |
| Chr09 | DiM_9-45 | 62.5 | 1 | SNP |
| Chr09 | Bng006 | 66.1 | 1 | RFLP |
| Chr09 | DiM_9-46 | 69.2 | 1 | SNP |
| Chr09 | DiM_9-47 | 69.8 | 1 | SNP |
| Chr09 | DiM_9-48 | 70.7 | 1 | SNP |
| Chr09 | DiM_9-49 | 71.4 | 1 | SNP |
| Chr09 | DiM_9-50 | 73.3 | 1 | SNP |
| Chr09 | Bng111 | 76.6 | 1 | RFLP |
| Chr10 | DiM_10-1 | 0 | 1 | SNP |
| Chr10 | DiM_10-2 | 1.7 | 1 | SNP |
| Chr10 | DiM_10-3 | 2.3 | 1 | SNP |
| Chr10 | DiM_10-4 | 4 | 1 | SNP |
| Chr10 | DiM_10-5 | 5 | 1 | SNP |
| Chr10 | DiM_10-6 | 6.4 | 1 | SNP |
| Chr10 | Bng200 | 8.2 | 1 | RFLP |
| Chr10 | DiM_10-7 | 11.2 | 1 | SNP |
| Chr10 | DiM_10-8 | 12.6 | 1 | SNP |
| Chr10 | DiM_10-9 | 13.6 | 1 | SNP |
| Chr10 | DiM_10-10 | 15.1 | 1 | SNP |
| Chr10 | DiM_10-11 | 17 | 1 | SNP |
| Chr10 | DiM_10-12 | 19.2 | 1 | SNP |
| Chr10 | Bng234 | 22.7 | 1 | RFLP |
| Chr10 | Bng068 | 28.9 | 1 | RFLP |
| Chr10 | DiM_10-13 | 34.1 | 1 | SNP |
| Chr10 | DiM_10-14 | 35.3 | 1 | SNP |
| Chr10 | Bng254 | 37.2 | 1 | RFLP |
| Chr10 | DiM_10-15 | 38.5 | 2 | SNP |
| Chr10 | DiM_10-16 | 39.3 | 1 | SNP |
| Chr10 | DiM_10-17 | 40.5 | 1 | SNP |
| Chr10 | DiM_10-18 | 41.4 | 1 | SNP |
| Chr10 | DiM_10-19 | 43.1 | 1 | SNP |
| Chr10 | Bng121 | 46.3 | 1 | RFLP |
| Chr10 | DiM_10-20 | 48 | 0 | SNP |
| Chr10 | DiM_10-21 | 48.4 | 1 | SNP |
| Chr10 | DiM_10-22 | 49.1 | 1 | SNP |
| Chr10 | DiM_10-23 | 55.2 | 1 | SNP |
| Chr10 | Bng218a | 57.9 | 1 | RFLP |
| Chr11 | DiM_11-1 | 0 | 1 | SNP |
| Chr11 | Bng076 | 2.1 | 1 | RFLP |
| Chr11 | Bng154 | 4.3 | 1 | RFLP |
| Chr11 | Bng078 | 6.5 | 1 | RFLP |
| Chr11 | DiM_11-2 | 9.3 | 1 | SNP |
| Chr11 | DiM_11-3 | 11.1 | 1 | SNP |
| Chr11 | DiM_11-4 | 12.9 | 2 | SNP |
| Chr11 | DiM_11-5 | 13.3 | 1 | SNP |
| Chr11 | DiM_11-6 | 14.1 | 1 | SNP |
| Chr11 | DiM_11-7 | 15.4 | 1 | SNP |
| Chr11 | DiM_11-8 | 16.7 | 1 | SNP |
| Chr11 | DiM_11-9 | 17.3 | 1 | SNP |
| Chr11 | DiM_11-10 | 19.7 | 1 | SNP |
| Chr11 | DiM_11-11 | 22.4 | 1 | SNP |
| Chr11 | DiM_11-12 | 23.1 | 1 | SNP |
| Chr11 | DiM_11-13 | 23.6 | 1 | SNP |
| Chr11 | DiM_11-14 | 25 | 1 | SNP |
| Chr11 | DiM_11-15 | 29 | 1 | SNP |
| Chr11 | DiM_11-16 | 32.3 | 1 | SNP |
| Chr11 | DiM_11-17 | 33.8 | 1 | SNP |
| Chr11 | DiM_11-18 | 35.1 | 1 | SNP |
| Chr11 | Bng253 | 37 | 1 | RFLP |
| Chr11 | DiM_11-19 | 38.2 | 1 | SNP |
| Chr11 | DiM_11-20 | 39.2 | 1 | SNP |
| Chr11 | DiM_11-21 | 42.2 | 1 | SNP |
| Chr11 | Bng192 | 45.5 | 1 | RFLP |
| Chr11 | DiM_11-22 | 46.7 | 1 | SNP |
| Chr11 | DiM_11-23 | 47.7 | 1 | SNP |
| Chr11 | DiM_11-24 | 50.1 | 1 | SNP |
| Chr11 | DiM_11-25 | 50.9 | 1 | SNP |
| Chr11 | DiM_11-26 | 51.6 | 1 | SNP |
| Chr11 | DiM_11-27 | 54.7 | 1 | SNP |
| Chr11 | DiM_11-28 | 55.9 | 1 | SNP |
| Chr11 | DiM_11-29 | 56.8 | 1 | SNP |
| Chr11 | DiM_11-30 | 57.7 | 1 | SNP |
| Chr11 | DiM_11-31 | 58.7 | 1 | SNP |
| Chr11 | DiM_11-32 | 59.3 | 1 | SNP |
| Chr11 | DiM_11-33 | 59.6 | 1 | SNP |
| Chr11 | DiM_11-34 | 61.6 | 1 | SNP |
| Chr11 | DiM_11-35 | 63.6 | 1 | SNP |
| Chr11 | DiM_11-36 | 64.6 | 1 | SNP |
| Chr11 | DiM_11-37 | 66.5 | 1 | SNP |
| Chr11 | Bng070 | 69 | 1 | RFLP |

NA: Not Applicable
