# Supplementary material for: Punctuated Distribution of Recombination Hotspots and Demarcation of Pericentromeric Regions in Phaseolus vulgaris L
Source: PLoS One. 2015 Jan 28;10(1):e0116822. doi: 10.1371/journal.pone.0116822 (PMC4309454; doi:10.1371/journal.pone.0116822)
Supplement: S2 Table — (DOCX) [file pone.0116822.s012.docx]

**Table S2. Primer sequences corresponding to mapped soybean-derived SNP markers**

| Marker | Forward | Reverse |
| --- | --- | --- |
| DGrpU | ATTTTGAGAATGGGGAAACG | GATGGAAACTCCCGTCAAAA |
| GG3.2SD1 | CACTGGCACTTCTCCATTCT | TTTGATAGATATGCAGTGATTGGAA |
| Hg1.1SDfr | CTGAAACCATGATCAAATTCAA | TCCGCTATCAGACCATTATCG |
