# Supplementary material for: Punctuated Distribution of Recombination Hotspots and Demarcation of Pericentromeric Regions in Phaseolus vulgaris L
Source: PLoS One. 2015 Jan 28;10(1):e0116822. doi: 10.1371/journal.pone.0116822 (PMC4309454; doi:10.1371/journal.pone.0116822)
Supplement: S3 Table — (DOCX) [file pone.0116822.s013.docx]

**Table S3. Chi-square test to detect randomness in marker distribution within defined intervals across the linkage map.**

| Marker intervals (cM) | Mean(μ) | *P* value |
| --- | --- | --- |
| 1 | 0.54 | <0.001 |
| 2 | 1.09 | <0.001 |
| 3 | 1.62 | <0.001 |
| 4 | 2.17 | <0.001 |
| 5 | 2.71 | <0.001 |
| 10 | 5.40 | <0.001 |
| 15 | 8.14 | <0.001 |
